# *Modeling in the Time of COVID-19:* Statistical and Rule-based Mesoscale Models

**DOI:** 10.1109/TVCG.2020.3030415

**Published:** 2021-01-28

**Authors:** Ngan Nguyen, Ondřej Strnad, Tobias Klein, Deng Luo, Ruwayda Alharbi, Peter Wonka, Martina Maritan, Peter Mindek, Ludovic Autin, David S. Goodsell, Ivan Viola

**Affiliations:** King Abdullah University of Science and Technology (KAUST), Saudi Arabia.; King Abdullah University of Science and Technology (KAUST), Saudi Arabia.; TU Wien and Nanographics GmbH.; King Abdullah University of Science and Technology (KAUST), Saudi Arabia.; King Abdullah University of Science and Technology (KAUST), Saudi Arabia.; King Abdullah University of Science and Technology (KAUST), Saudi Arabia.; Scripps Research Institute, US.; TU Wien and Nanographics GmbH.; Scripps Research Institute, US.; Scripps Research Institute, US.; King Abdullah University of Science and Technology (KAUST), Saudi Arabia.

**Keywords:** molecular visualization, mesoscale modeling

## Abstract

We present a new technique for the rapid modeling and construction of scientifically accurate mesoscale biological models. The resulting 3D models are based on a few 2D microscopy scans and the latest knowledge available about the biological entity, represented as a set of geometric relationships. Our new visual-programming technique is based on statistical and rule-based modeling approaches that are rapid to author, fast to construct, and easy to revise. From a few 2D microscopy scans, we determine the statistical properties of various structural aspects, such as the outer membrane shape, the spatial properties, and the distribution characteristics of the macromolecular elements on the membrane. This information is utilized in the construction of the 3D model. Once all the imaging evidence is incorporated into the model, additional information can be incorporated by interactively defining the rules that spatially characterize the rest of the biological entity, such as mutual interactions among macromolecules, and their distances and orientations relative to other structures. These rules are defined through an intuitive 3D interactive visualization as a visual-programming feedback loop. We demonstrate the applicability of our approach on a use case of the modeling procedure of the SARS-CoV-2 virion ultrastructure. This atomistic model, which we present here, can steer biological research to new promising directions in our efforts to fight the spread of the virus.

## 1 Introduction

All living organisms on Earth share a common complex, hierarchical structure. At the lowest level of the hierarchy, biomolecules such as proteins and DNA perform all of the basic nanoscale tasks of information management, energy transformation, directed motion, etc. These biomolecules are assembled into cells, the basic units of life. Cells typically are surrounded by a lipid bilayer membrane, which encloses several thousand different types of biomolecules that choreograph the processes of finding resources, responding to environmental changes, and ultimately growing and reproducing. Most familiar organisms, such as plants and animals, add an additional level to this hierarchy, with multiple cells cooperating to form large, multi-cellular organisms.

Viruses are pared-down versions of living organisms, with just enough of this hierarchical structure to perform a targeted task: to get inside a cell and force it to create more copies of the virus. Viruses are typically comprised of some form of nucleic acid (RNA or DNA) that encodes the genome and a small collection of proteins that are encoded in this genome, which together form the molecular mechanism for finding cells and infecting them. Some viruses also include a surrounding envelope composed of a lipid bilayer membrane that is acquired as the virus buds from an infected cell.

Effective computational methods are available for modeling and visualizing the biomolecular components of cells and viruses. Atomic structures of over a hundred thousand biomolecules are available at the Protein Data Bank wwpdb.org) [3], and decades of research and development have generated a comprehensive toolbox of simulation, structure prediction, modeling, and visualization tools to utilize and extend this data [53,57]. However, modeling and visualization of the full hierarchical structure of living organisms—from atoms to cells—is a field still in its infancy, limited largely by the size and complexity of the hierarchy and its many interacting parts. Modeling and visualization of the cellular mesoscale—the scale level bridging the nanoscale of atoms and molecules with the microscale of cells—is necessarily an integrative process, since there are no existing experimental methods for directly observing the mesoscale structure of cells [22]. Mesoscale studies integrate information from microscopy, structural biology, and bioinformatics to generate representative models consistent with the current state of knowledge. Challenges that are currently limiting the integrative modeling pipeline include (a) finding and curating disparate sources of data, and (b) constructing and visualizing intuitive 3D models of this size and complexity with reasonable user and computational effort. This latter challenge is addressed in this paper.

The central idea behind our rapid modeling approach for mesoscale models is to take advantage of the repetitive structure of the hierarchy of living systems. We model the structural characteristics of a small representative collection of structural elements, which are then assembled into the entire cellular or viral system through a set of learned rules that guide placement and interaction of the component elements. These rules are specified directly through 3D interactive modeling, instead of indirectly through a rule-definition syntax. In this way, we can reduce the burden on the users, provide them with an intuitive modeling interface, and automatically generate instances of the full model comprised of a huge number of interactive component elements. In cases where the model needs to be further fine-tuned or new information needs to be incorporated, the construction rules are revised in 3D and new models are generated that incorporate the latest revisions. If structural evidence is available in the form of electron microscopy (EM) images, our system determines basic structural properties from these images while requiring few inputs from the user.

We demonstrate this rapid modeling method for integrating data from electron microscopy with structural information for the novel coronavirus SARS-CoV-2. The generated models can be used for exploring the diversity of structure and analyzing the detailed arrangement of spike glycoproteins on its surface.

## 2 Related Work

Modeling of geometric representations of molecules has been driving scientific visualization and computer graphics research for several decades. In the late seventies, Richards developed a geometric representation of molecular surfaces that characterized their area [60], which was further popularized by Connolly [14]. Over the years, many geometric construction algorithms for *molecular surfaces* have been developed, notably Reduced Surface [61], blobby objects [5,55], or *α*-shapes [17], to name a few. These algorithms are typically well parallelizable on multiprocessor systems [71] or on modern GPUs [12,29,39] et al., and nowadays scale up to interactive rates of huge atomistic models thanks to, for example, visiblity-driven rendering strategies [7]. Simplified representations such as van-der-Waals space filling molecular models can be interactively constructed and visualized to represent scenes with up to a billion atoms [19,37,43] et al. by making use of various acceleration strategies, such as the procedural impostors [68], adaptive level-of-detail tesselation [40,47], or hybrid particle-volumetric representation [62].

The geometric representations of the molecular structures described above have been mostly concerned with modeling protein macromolecules. Recently, dedicated approaches for modeling large lipid membranes have been developed [4,15], as well as new approaches to modeling fibrous macromolecules such as the 3D genome. Halladjian et al. presented an approach to construct and visualize a multi-scale model of interphase chromosomes [25]. Procedural modeling of the backbone of linear polymers like RNA or DNA is typically approached by concatenating building blocks with processes like a random walk. A random walk produces a sequence of points where the location of each generated point is dependent on its predecessor. While this process leads to plausible models and is able to incorporate measured characteristics like the stiffness, it is hard to control and guide to specific points. Klein et al. [38] propose a parallel algorithm for constructing a 3D genome sequence, which builds on the midpoint-displacement concept. We utilize this approach for calculating the path for nucleic acids.

The above methods model molecular geometry based on some underlying well-defined structure. The technique presented in this paper is primarily concerned with interactive 3D modeling of molecular assemblies, which is inspired by methodologies developed in graphics research. In particular, our methods build on *rapid 3D modeling*, which is a process where the author specifies the desired 3D model through a minimal amount of user interactions. The algorithm or determined statistical model then constructs the geometric model by preserving user-defined constraints. There are two dominant strategies for achieving rapid 3D modeling.

The first methodology, known as *sketch-based modeling*, allows the user to specify certain geometric details directly in the scene. A good example for sketch-based modeling is the Teddy system presented by Igarashi et al. [31]. Here the user only specifies a 2D contour of an object and a 3D geometry is generated using the contour inflation approach [76], which we also utilize. An interesting recent trend is to control a deep learning model using sketches, e.g., for modeling terrains [23], faces [58], or buildings [52]. Utilization in the sciences can be exemplified through modeling advanced geological concepts and phenomena [41,48] or for creating quick molecular landscapes for communicating to peers or a broader audience. For example, Cell-PAINT [21] is a system that allows users to create 2D mesoscale animated illustrations on the web interactively by using molecular palette and system-defined rules. Users can use pre-defined behavior of components of the mesoscale model. These works result in approximate *sketches* of complex scientific scenarios and make use of rules that are algorithmically defined within the system.

The second approach is known as *procedural modeling* and its basic idea is that the geometric structure is defined indirectly by specifying the rules and the parameters of these rules. The rules are then used when executing procedural construction of the 3D scene geometry, often without any direct geometric input from the user. The *rapid* modeling aspect is achieved through the quick setting of a few parameters that can serve as sufficient input for massively large scenes. Procedural modeling has a long tradition in computer graphics [16]. It is frequently used for modeling large environments that *look* plausible. Examples are models of vegetation [59], cloudscapes [75], roads [20], street networks [54], and buildings [46,64].

Procedural modeling has been utilized in sciences beyond visually plausible modeling to create scientifically accurate models. Biologists can recreate mesoscale systems using procedural modeling methods, based on constraints from nanoscale and microscale measurements. Johnson et al. [32] have developed a system called cellPACK that takes a recipe as an input, which is a description of how structures should be positioned in the organism model. A packing algorithm then iteratively places the macromolecular building blocks into different compartments of the model. This compartment is described by a discretized distance volume, which is packed and updated in a sequential manner. Currently, on a desktop workstation, such a packing process takes several minutes up to hours, to pack a representation of the HIV virion that is 100 nm in diameter. However, the specification of the recipe is a human-readable textual rule definition that relies on accurate specification from the user.

## 3 Statistical and Rule-based Modeling

**The requirements** that guide the design of our approach are scientific relevance, intuitiveness, rapidness, reusability, revisionability, and controlled precision. Scientific relevance requires a model to be an abstraction of reality that incorporates all components of reality known at that particular time. For unknown information, the most accepted hypothesis may be incorporated in the model. Intuitiveness requires the target users, i.e., structural biologists, to be able to express their ideas about a given structure effortlessly in, what is for them, a natural way. Rapidness requires the process be completed at a fast pace and reusability requires the ability to reuse previously modeled components in other assemblies. Revisionability allows users to revise a detail without the necessity for manual remodeling of the entire assembly and controlled precision allows users to create assemblies with varying degrees of precision in structure alignment.

As a **target user group**, we focus on modelers who are structural biologists and who know or study a particular structure holistically and aim to integrate individual elements to form the entire structure. This target group has neither strong programming skills nor a formal computer science background. Their envisioned ambition is to create a model that cannot be created with conventional molecular modeling methods, based on Newtonian physics simulation solvers. The modeling outcome is primarily an externalization of modelers’ understanding of the ultrastructural assembly, which can be used for communication, hypothesis generation and validation, or even serving as input for classical simulation-based methods.

The proposed 3D modeling technique features both sketching and procedural modeling philosophy for the rapid creation of scientifically relevant mesoscale models. The **foundation** of the technique is an intuitive visual-programming strategy, where the modeler expresses possible assembly configurations. Our technique applies a *copycat* principle to rapidly complete the model driven by the expressed rule-set. This strategy replaces methodologies that previously relied on domain-specific languages for formulating such modeling rule-sets that were not well accepted by the structural biology community. This overarching approach is complemented by multiple novel and existing supportive technologies that allow for completion of scientifically accurate mesoscale models.

Unlike, for example, cellPAINT [21], cellPack [32], and instant construction mesoscale assembly techniques [37,38], which are perhaps the closest techniques for **comparison**, the proposed approach is initially rule-free and all rules are specified by the user, incorporating characteristics extracted from imaging data whenever possible. In the above-mentioned techniques, all the rules are formulated within the algorithm, or are pre-defined for each structure. Moreover, in our case, the rules can be defined at a wide range of modeling precision, from a precise to a more approximate placement. Our rule design space is open; many simple rules are generated first, then can be combined in a construction of more complex structural elements, and obsolete concepts can be revised into new rules and effortlessly reapplied. The established rules can be stored as structural templates that can be shared among users.

In the context of scientific data visualization, the modeling methods that are employed need to provide suitable representations for hypothesis generation, testing, or even in the simulation of stability and dynamics. To be able to create models that are **scientifically relevant**, our modeling framework needs to allow for versatile structural arrangement specification and needs to support integration with acquired evidence from microscopy data. These requirements differ from existing procedural modeling methods. For example, L-system models [42] are typically topological trees and the procedural models are based on growing plants according to the tree structure and architectural models are generated by top-down subdivision with elements in regular arrangement. Mesoscale biological models are a different case. In this novel scenario, many elements are in relation to each other and interact with each other. The arrangement becomes much more irregular than in architecture, while the statistical variation is specified in a controlled manner.

**Mesoscale biological structure** is typically characterized at the nanoscale by its molecular composition, where molecular structure can be either measured or simulated. The microscale is characterized from microscopy images or tomographic volume reconstructions of the entire entity. Rough shapes of the macromolecules can often be observed in these image data, so typically several hypotheses can be formulated about the specifics of the assembly. Usually the membrane boundaries and associated proteins are more recognizable than the soluble assemblies inside the membrane. Therefore, we model the membrane information based on image data and the information inside the membrane is characterized through interactive 3D modeling using structural rules.

In our work, we concentrate on the **extraction of membrane** outlines or contours that are often apparent in microscopic images. First, a handful of membrane contours are traced by the user. These contours are co-registered to analyze their variation. Such representation is statistically captured so that many new contours, similar to the input samples, can be generated. Based on the contour information, a three-dimensional virion geometry is estimated that matches the contour shape. Resulting mesh representations of virions are populated with molecules bound to the membrane according to the observations in the images. We characterize the molecular distribution around the contour and estimate a corresponding distribution for the entire virion surface.

Once the information from the images is incorporated into the mesoscale model, further modeling of elements that are not directly observed in image data is used to complete the model. Several hypotheses can be generated to express what a biologist considers as a valid assembly configuration. The modeling proceeds through an interactive **3D rule specification** process, where the modeler expresses certain spatial relationships on exemplary structural representatives. An interactive 3D visualization shows how this rule is applied for the corresponding molecular population. Based on the instantaneous visual feedback, the modeler can revise previous inputs to obtain the desired assembly. In this stage, hierarchical relationships can be utilized for expressing the rules that define distance and orientation distributions among molecular instances. The scene population is corrected by collision handling so that a valid molecular scene results from the application of the rules.

**Fig. 2.**
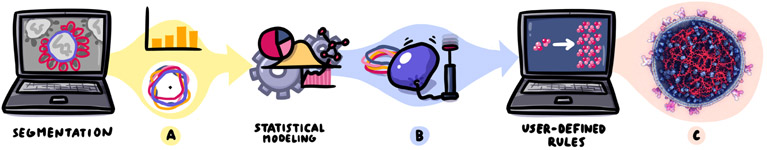
An overview of our mesoscale modeling pipeline. First, the user specifies the segmentation of the membrane outlines (contours) and visible membrane-embedded proteins. Contours and a histogram of amounts of proteins within individual parts of the membrane (A) are used for statistical contour modeling that is inflated into a 3D mesh and populated with membrane proteins (B). Subsequently, the user specifies the rules outlining how invisible proteins should be placed within the 3D model. The output is the model (C), which can be iteratively refined by modifying the rules.

An overview of the modeling process and the steps described above is shown in Figure 2. The following sections (Section 4, Section 5) describe the technical details of our approach.

## 4 Imaging-Driven Shapes and Distributions

At the beginning of our approach, EM images are segmented. A set of contours together with a distribution of surface proteins is estimated. Then, a mesh representing the shape of the virion with every triangle evaluated by a probability for surface protein placement is generated. The detailed description follows.

### 4.1 Image segmentation

**Fig. 3.**
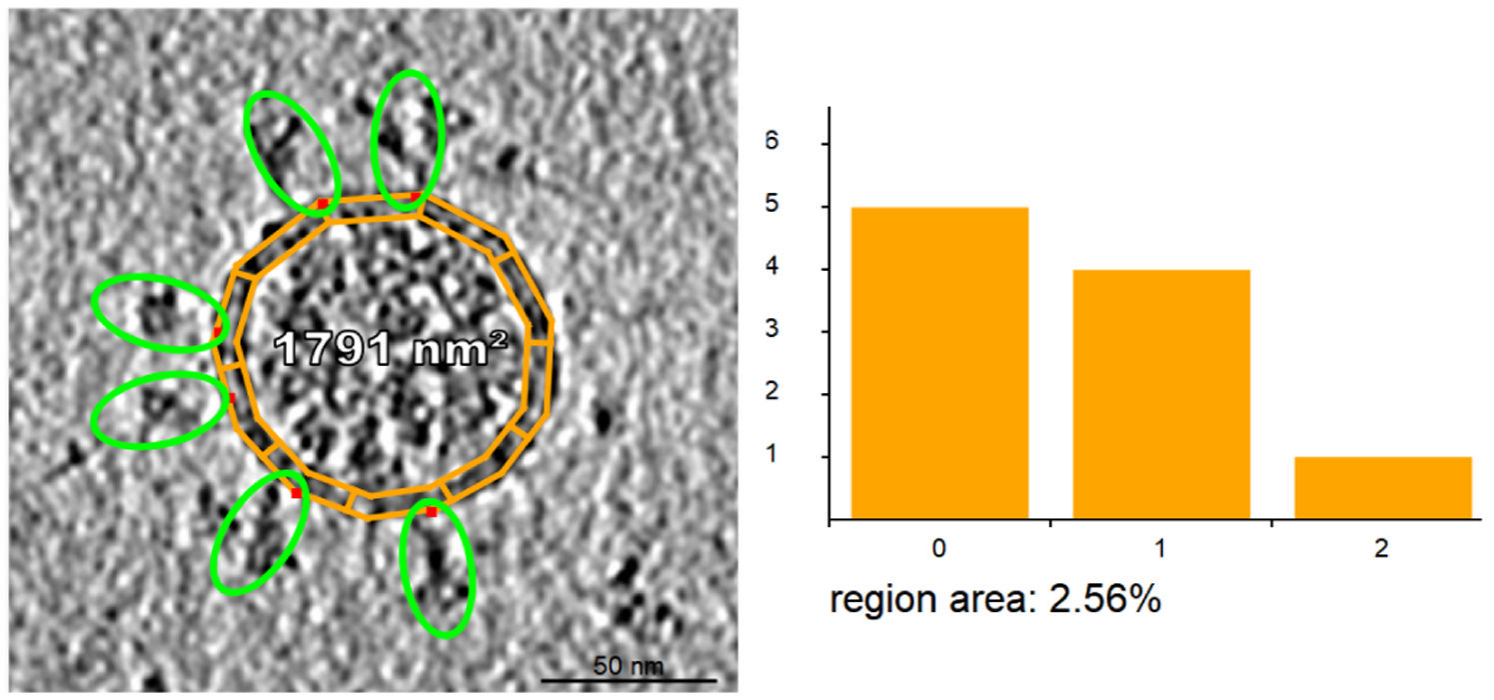
Input electron microscopy [34] image after segmentation. Left: The contour with a band is created. Right: The histogram representing the number of spike proteins per contour band region.

For rapid processing of electron microscopy images, we implemented a segmentation tool that produces the input for determining the contour of the membrane and the protein distribution on the membrane. The user creates an outline, the outer contour of the virion, and places small elliptical proxy objects representing proteins scattered over the surface of the virion. The major axis of the ellipse is aligned with the main axis of the protein. We perform this quick feature extraction for all proteins that are close to the cross-section or silhouette of the membrane, as shown in Figure 3. These proteins naturally are not exactly on the contour, but they are located close to the contour within a certain surface band. To characterize this band, an inner contour is specified. The user can easily specify the thickness of this band on which the marked surface proteins are located. Next, a distribution of the surface proteins on the membrane band needs to be estimated. For this, we subdivide the band into equally sized surface patches and count the amount of proteins associated with each patch. To characterize the distribution of the proteins from what we see on the membrane contour, we store the per-patch protein counts in a histogram. This gives us a distribution function of the amounts of protein per patch area, which we use when we populate membrane-protein instances on the 3D model of the membrane. Note that we do not count spike proteins of the virion in the 2D image to project this number to the 3D surface. Although this estimation exists, the final amount of surface proteins is decided by the user in the later phase. The main outcome of this phase is the contour of the virion and the probability distribution function. This approach has been inspired by methods used in a recent publication characterizing membrane proteins [34], which used the highlighting approach for indicating spike proteins. Instead of data *analysis*, we follow their intuitive specification method for model *synthesis.*

Once we obtain a set of membrane contours extracted from multiple virions, we then use them for generating new distinct contours that have similar characteristics. For that, we need to register all contours into a common coordinate system. We do this by fitting an ellipse to each contour and then translating and rotating the contours such that the approximating ellipses are in canonical form. We assume that all contours can be approximated by an ellipse. To parametrize an ellipse, we use the two focal points and the semi-major length. A point **p** is on the ellipse if and only if:

(1) $$\|p-c_{1}{\|}_{2}+\|p-c_{2}{\|}_{2}=2a$$

where **c_1_** = (*c*_1_.*x*, *c*_1_.*y*), **c_2_** = (*c*_2_.*x*, *c*_2_.*y*) are the focal points and *a* is the semi-major length. To fit an ellipse to a set of data points $p_{i}=(p_{i\cdot }x,p_{i\cdot }y{)}_{i=1}^{n}$, we pose the problem as an optimization problem [81]:

$$\underset{c_{1},c_{2},a}{\text{min}}\frac{1}{n}\sum_{i=1}^{n}(\|p_{i}-c_{1}{\|}_{2}+\|p_{i}-c_{2}{\|}_{2}-2a{)}^{2}$$

**Fig. 4.**
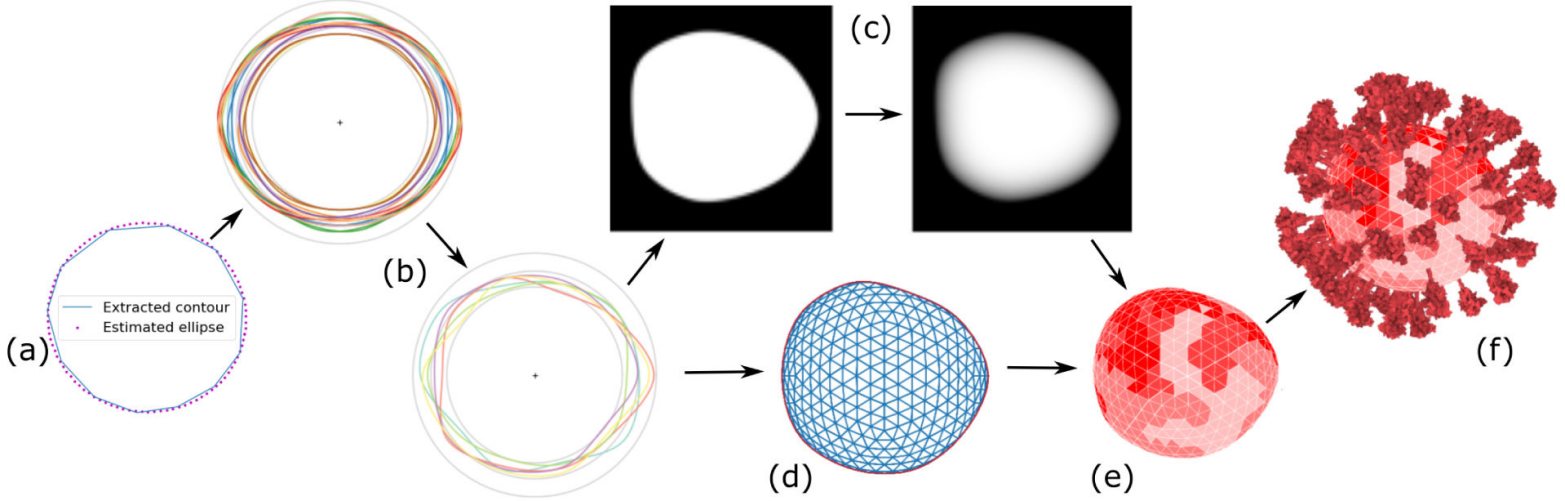
Statistical contour modeling for virion mesh generation: The contour is approximated by an ellipse that is used for bringing all contours into a canonical form (a). Statistical contour model is generated from a set of contours and new contours can be generated (b). A newly generated contour is rasterized for contour inflation (c). A two dimensional mesh is generated (d) which is then inflated into the 3D mesh with probability distribution assigned to its triangles (e). Spike proteins are populated (f).

This objective function has a global minimum at infinity. When the two focal points move to infinity and the semi-major length tends to infinity, the value of this function approaches zero. We therefore add an L2 regularizer to avoid the undesirable global minimum at infinity:

(2) $$\underset{c_{1},c_{2},a}{\text{min}}\left[\frac{1}{n}\sum_{i=1}^{n}(\|p_{i}-c_{1}{\|}_{2}+\|p_{i}-c_{2}{\|}_{2}-2a{)}^{2}+\frac{\lambda }{n}({c_{1}}^{2}+{c_{2}}^{2}+(2a{)}^{2})\right]$$

where *λ* is a tuning parameter. In the initialization, *a* is initialized as the mean of the distance from the data points to the mean of all data points **p**_*μ*_, $c_{1}=(-\frac{a_{max}}{2},0)$, $c_{2}=(\frac{a_{max}}{2},0)$, where *a*_*max*_ is the largest distance from a data point to **p***_μ_*. After initialization, the penalized objective function Equation 2 can be solved by gradient descent. The obtained ellipse has semi-major length **a**, semi-minor length **b**, center **c**_**e**_ and angle of rotation *θ*_**e**_. The example of estimated ellipse can be seen in Figure 4(a).

To register the contours into a common coordinate system, we estimate the translation and rotation based on the approximating ellipse. First, the segmented contour is translated to the origin **O**(0,0) by translation vector **t** = −**c**_**e**_. Then, the segmented contour is rotated by angle −*θ*_**e**_.

Each contour in the set of contours is reparameterized by *N*_*p*_ points **p_i_**. Each point **p_i_** is defined by an angle ***θ*_i_** and a distance *r*_*i*_ = ∣**Op_i_**∣ in a polar-coordinate system. To increase the accuracy of the generating step, we generate several other orientations from the contours. We create three augmented contours for each contour. The first augmented contour is obtained by a rotation with angle *π*. The second and third augmented contours are obtained by flipping the original and the rotated contour through the x-axis. Four contours - the original contour with three augmented contours - are used for the next step. To generate a new contour from the contours, we compute a per-angle one-dimensional normal distribution by casting a ray from the origin **O** in all *θ*_**s**_ directions and intersecting all contours with this ray. To the *N*_*p*_ intersection (∩) points **p**_∩_**i**__, we fit a normal distribution for *N*_*p*_ **r**_∩_**i**__ = ∣**Op**_∩_**i**__∣ with mean *μ*_**s**_ and standard deviation σ_**s**_ as parameters. We also truncate the normal distribution to the minimum and maximum distance values in the data. From these parameters, we perform rejection sampling [11] of the truncated normal distribution of **r**_∩_**i**__ for each angle *θ*_**s**_ (*θ*_**s**_ ∈ [0,2*π*]). Finally, we interpolate the points using Catmull-Rom splines to create a new contour. The input contours and a number of generated contours are shown in Figure 4(a, b). The algorithm is listed in Algorithm 1 and Algorithm 2 and can be found in Supplementary Material.

### 4.2 Virion shape generation

From the generated contour, our next step is to generate a membrane, which is a three-dimensional ellipsoidal *potato*-like object. We model the object based on three principal dimensions, *d*_1_ ≥ *d*_2_ ≥ *d*_3_ of an ellipsoid, and characterize it by two aspect ratios, namely as elongation index *EI* = *d*_2_/*d*_1_ and flatness index *FI* = *d*_3_/*d*_2_ [69]. *EI* can be determined from the contour. *FI* is defined by the user and then used for the determined *d*_3_. Next, we need to extrude the contour into three dimensions. For this task, we employ the standard contour inflation method from sketch-based modeling [76]. To assign a depth (*z*) value to all points on the three-dimensional object, we proceed as follows. First, we make a binary mask from the contour so that 0.0 is assigned to the outside of the shape and 1.0 to the inside of the shape. Then, we apply a cascade of Gaussian filters (with radius 32, 16, 8, 4, 2, 1) on the image mask. After each smoothing pass, the resulting image is multiplied with the original image mask, so that all pixels that are outside the contour are again set to zero. The resulting image is used for assigning the depth values symmetrically on both subspaces partitioned by the contour plane of *z* = 0 (see Figure 4 (c)). The next step is to create the three-dimensional object represented by a triangular mesh. We create a 3D sphere with approximately equally sized triangles, where the radius is the largest radius from all contour points to the origin **O**. After that, we project this mesh onto the *z* = 0 contour plane. This projected mesh is distorted to the shape of the contour. The example 2D mesh backprojected onto the contour plane can be seen in Figure 4 (d). Finally, for each mesh point, we extrude its *z*-coordinate to *inflate* the contour. The *z*-coordinate value of each point of the mesh is calculated as a multiplication of half of *d*_3_ with the corresponding pixel value (with the same *x, y* coordinates) from the previously calculated depth image in Figure 4 (c).

In the image segmentation phase, a band around the contour is created, subdivided into ten equally-sized regions, and the amount of membrane proteins belonging (i.e., within close proximity) to the region is evaluated. We determined this value of ten regions based on our experiments. Lower numbers correspond to more uniform the target distribution on the 3D mesh, and with larger numbers, the higher the probability distribution would be concentrated to small areas of the 3D mesh only. With this construction, we obtain the distribution of membrane proteins per given area because we know the area of a single region. We use this distribution for populating the membrane proteins on the triangular mesh. The membrane protein density of triangles is computed in the following way. First, the 3D mesh is partitioned into approximately same-sized triangular patches. The size of the patch is determined by the size of the area of segments of the bands from the 2D contour. Afterwards, every patch is associated with one value from the above distribution. We use random sampling of the distribution. Then we distribute the number of membrane proteins among the triangles that belong to the current patch. Examples of generated 3D meshes with associated protein counts per triangle can be seen in the bottom right of Figure 18, along with the examples of membranes with the membrane proteins.

## 5 Interactive 3D Rule Specification

The second part of our approach is used to populate the model with biological elements that are placed in relation to other elements in the virion. While some of these elements cannot be clearly seen in EM images, their structural information is generally understood, or at least there is a hypothesis on the structural organization. For example, a protein can be in a spatial relation (position, rotation) with another protein. The rules encode how new elements can be placed based on the geometry of already existing elements.

We create a three-dimensional *model* that consists of a set of *elements*. Our interactive procedural modeling approach organizes the elements in a tree. An element consists of the following: 1) A name to identify the element, e.g., to select an input element to a rule. 2) A type that can be either auxiliary or instance. An auxiliary element will be invisible in the final model and an instance will be visible. We often refer to an auxiliary element as *skeleton*. 3) The element geometry that can be either a polygonal mesh, a poly-line, or a set of points. Sometimes the geometry is only a single polygon, line segment, or point. 4) A bounding sphere that consists of a local coordinate system used to position the geometry in the world coordinate system, an orientation vector and three scaling factors to determine the size of the element.

**Fig. 5.**
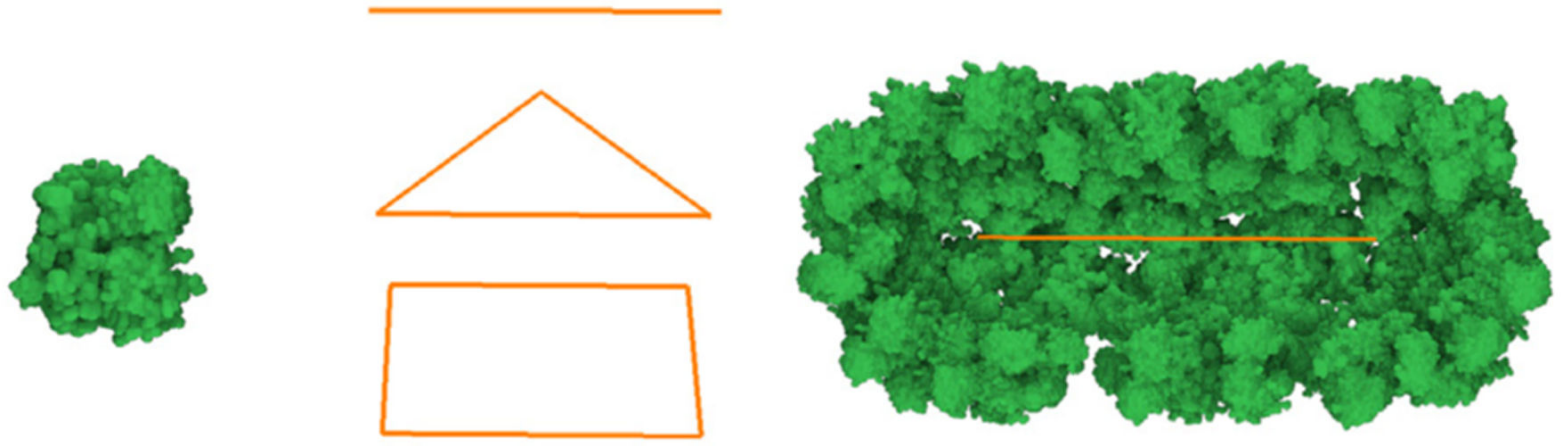
Illustration of element geometry. Left: protein instance from a database. Middle: a line segment, triangle, and rectangle. Right: an arrangement of protein instances around a line segment.

We use a library of structural models, for example proteins, in our framework. Many of these models are freely available on the internet. The most common form in which they are distributed is a list of atoms where the type and position of each atom is specified. Conceptually, we could convert these descriptions into 3D meshes, but we typically keep them in a different representation (e.g., set of spheres) for faster rendering. We also assign an identifier *G_id_* to them. These identifiers will be used in the rules to specify the geometry of elements. We also use a library of elementary meshes, such as single polygons, a tetrahedron, or an icosahedron that prove to be useful as an auxiliary geometry. See Figure 5 for an illustration of example geometries.

The main concept is based on the creation of a model from elements using rules. The function of a rule is to identify an element in the current model and to create one or multiple elements either as children or as siblings in the derivation tree. In contrast to other popular procedural modeling systems, such as [46,59] for example, our rules are not described by a script-like language, but they are designed and executed in an interactive editor. The user can interact with elements in a 3D visual-programming environment, e.g., positioning and rotating elements using a virtual gizmo tool. In the following, we will describe the most important concepts and parameters. We plan to release the executable and detailed UI documentation upon publication of this work.

### 5.1 Creation of the model

The creation of a model starts with an auxiliary root node of the tree and an empty model. The elements are placed by processing the specified rules and rule groups in a sequence. To preview the effect of rules on the whole model (or for example only on its part), the user has full control of the rule execution and can execute all rules at once or execute rules step by step, and perform interactive edits between the execution of rules. Furthermore, if the result is not as expected or a detailed part of the model is about to be solved, the user can undo rules or even partially undo rules. The rule is partially undone if it is reverted on a selected subset of elements from all elements to which the rule is applied. The rules take elements currently presented in the scene (identified by their name) as input and generate zero, one, or multiple new elements.

To create various distributions of elements, the rules can be organized into groups. If rules *r*_*i*_ are placed in a group, they can be either applied in an alternating manner, or rules can be selected randomly among the set of rules in the rule group according to their probability *r*_*i*_. *probability*.

Several geometric parameters (for example a distance or angle between two structures) can be specified as constants or as probability distributions. A probability distribution can be modeled by combining Gaussian and uniform functions as building blocks. The values are automatically normalized so that their sum integrates to one.

In all the rules, several rotational variants can be used to specify a transformation. Currently implemented variants are: user-defined rotation, random rotation, normal vector orthogonal to a parent element normal vector, and element normal vector aligned to a parent normal vector. Moreover, these rotations can be extended by user-specified yaw, pitch, and roll distributions that represent a deviation in rotation in the respective axis.

An important part of our approach is collision detection. We implemented an octree accelerated method. A naive collision detection algorithm turned out to be unusable due to the amount of elements in the model (~200000 element instances). We use an octree with four levels of subdivision. Every element in the scene is assigned a bounding sphere. This bounding sphere can be additionally scaled by the user to better approximate the object when the object is long but thin. Although this can lead to overlapping of elements, the property can be exploited, for example, in cases where string-like elements are placed in a plane close to each other. The user can control how close elements can be before creating a collision. Once a new element candidate is generated, all leaf octants of the tree intersecting the element’s bounding sphere are fetched and used for collision detection. If there is no collision, the element is created. Otherwise, the element is not created. To avoid rules that are not terminating because of collisions, we employ a parameter *collisions*_*max*_ to specify the maximum number of consecutive detected collisions. If that number is reached, a rule is terminated.

The specifications of all rules are stored in a file. Thus, the user can create a library of rules that can be then re-used as templates to build other mesoscale models.

### 5.2 Type of rules

We identify and implement four main classes of rules: parent-child, siblings, siblings-parent, and connection rule.

In the **parent-child rule**, new child elements are added to a parent element with name *Name_in_* given as input. We employ two types of rules, called the distance rule and relative rule.

**Fig. 6.**
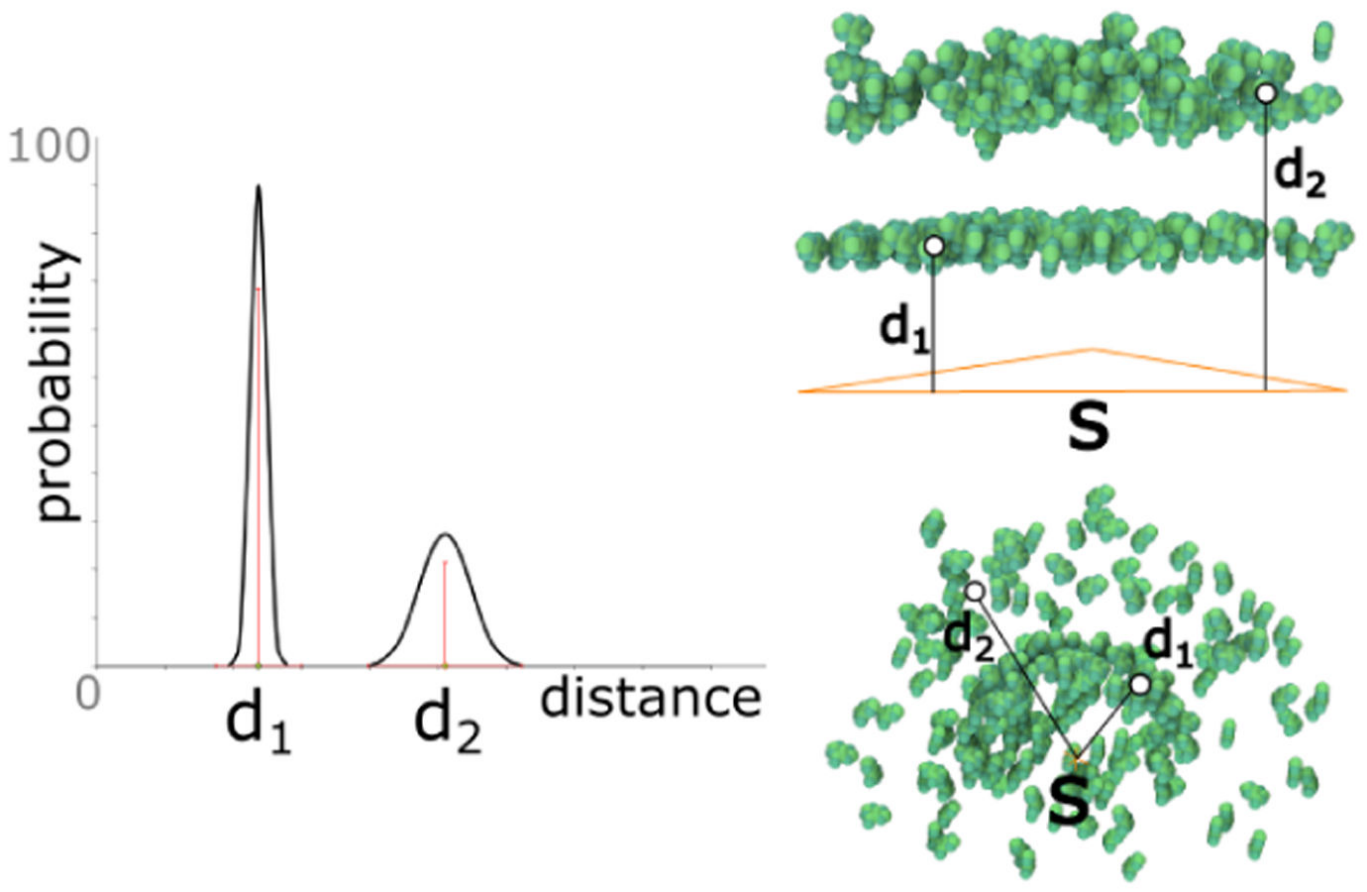
Distance rule. Left: The definition of a probabilistic distance function. Top right: Application of the rule to a triangular skeleton. Bottom right: Application of the rule to a point skeleton.

The main purpose of the *distance rule* is to create new elements at a specified distance to the parent. The distance *d* can be either a constant or modeled probability distribution that is sampled each time a new element is created. To determine the position of the new element, a random point on the parent geometry is generated and translated along the normal vector according to the (sampled) distance *d*. Another parameter determines if the translation happens along the positive normal direction, negative normal direction, or randomly selected among the two. In Figure 6 left, we illustrate a probability function that is modeled as a combination of two Gaussians with mean *d*_1_ and *d*_2_. The Gaussian around *d*_1_ has a higher weight than the Gaussian around *d*_2_. The resulting population of elements using a triangle and a point skeleton as parent are presented in Figure 6 middle and right, respectively.

**Fig. 7.**
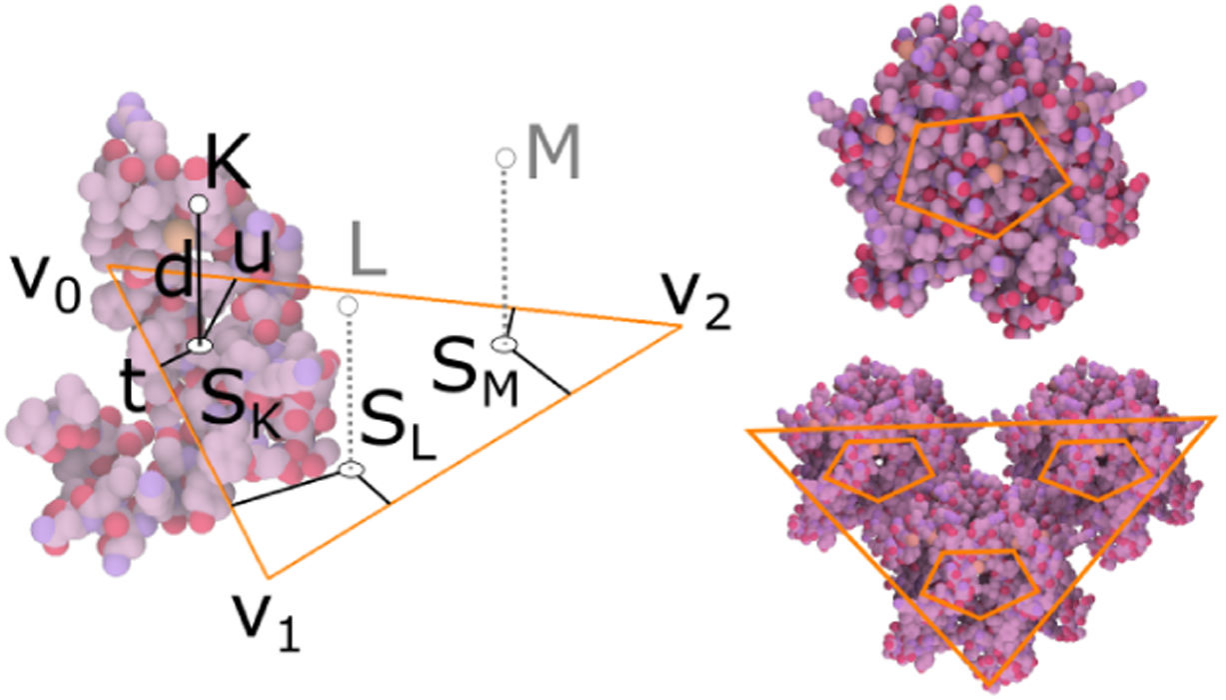
Relative rule. Left: Illustration of a relative rule created on a triangular skeleton. Top right: The same rule applied to a pentagonal skeleton. The result is a pentameric structure. Bottom right: The pentagonal skeleton model is bound to a triangular skeleton.

The *relative rule* specifies the location of new elements with respect to a vertex of a polygon of the input element. For example, in Figure 7 left) a position *K* is specified by the user and subsequently encoded with respect to vertex *v*_0_. The position is computed by the parameters *t*, *u* that specify the distance from the edges connected to *v*_0_ and the distance *d* along the normal of the polygon. The parameters *t*, *u* specify the location of *S_K_*, the closest point on the polygon. From these rule parameters, the corresponding positions *S_L_* and *S_M_* can be found inside the triangle, and new elements are placed in the points *L* and *M* that are at the distance *d* along the normal vector from positions *S*_*L*_ and *S*_*M*_, respectively. The rule created from the previous process can be transferred and applied to any polygon, e.g., to create a pentamer (an entity composed of five sub-units where each unit is placed in a vertex of the pentagon), as shown in Figure 7 middle. The example in Figure 7 right shows two subsequent applications of the rule to model the structure of a viral capsid. First, the relative rule is used to create three pentagon elements as children of a triangle element. Second, proteins are created as children of each of the pentagons.

**Fig. 8.**
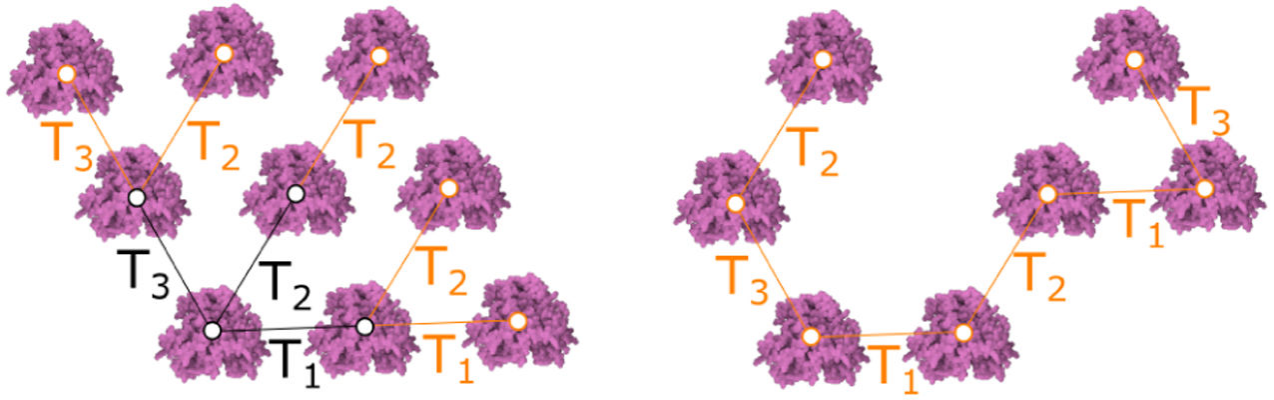
Siblings rule. Left: All three transformations are applied in every iteration. Right: One transformation is selected randomly per iteration.

The **siblings rule** creates new elements and adds them as siblings to the same parent in the tree. The most important parameter of the siblings rule is a set of transformations *T_i_*. These transformations are typically a combination of translation and rotation. Each transformation also has an associated probability *T_i_*.*prob*. To apply the rule, a transformation *T_i_* is selected according to the probabilities *T_i_*.*prob*. Then, a new element with name *Name*_*out*_ is generated by transforming the coordinate system of the input element *Name*_*in*_ and setting the geometry as specified by the identifier *G*_*id*_. A parameter *T*_*num*_ determines how many transformations will be selected. If *T*_*num*_ is equal to the number of transformations, all transformations will be selected and the probabilities will be ignored. The rule is invoked recursively for newly created elements. Identical to previous rules, the parameter *count*_*max*_ determines how many elements are inserted. In Figure 8 left, three different transformations of a single type element *T*_1_, *T*_2_, *T*_3_ were created. The user can generate these transformations interactively. In this example, an additional five instances were generated recursively (for *T*_*num*_ = 3). In the example Figure 8 right, *T*_*num*_ = 1 to showcase the random selection and application of transformations.

The **siblings-parent rule** is an extension of the siblings rule. The user specifies a transformation to the sibling element with name *Name*_*in*_, as before. After applying the transformation, the new element *Name_out_* is snapped to a given distance *d* from the parent shape of *Name*_*in*_. This distance preservation acts as a correction factor. The main benefit of this rule is that the user who wants to distribute elements in a circle around a point or a spiral around a line segment does not have to precisely measure the angle and translation.

**Fig. 9.**
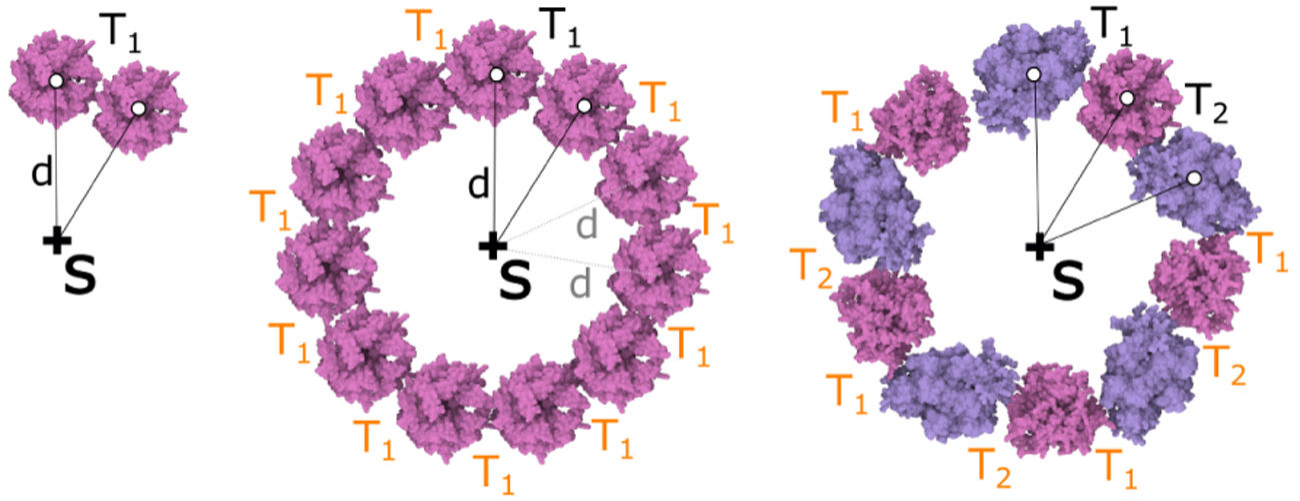
Siblings-parent rule. Left: Creation of a rule. Middle: Application of the rule. Right: Two rules applied alternatively to place two different elements in a circle.

In Figure 9 left, the parent shape is a point labeled *S* and the input element is labeled *K* and the newly generated element is labeled *L*. The transformation *T*_1_ consists of a translation. In subsequent applications of the rule, the distance *d* to the skeleton *S* is preserved (see Figure 9 middle). The user can specify a group of rules that can be applied in iterations. In Figure 9 right, two rules with transformations *T*_1_, *T*_2_ on two different element types were created and applied.

The **connection rule** is designed for creating string-like structures that pass through a given set of 3D points. These points typically represent positions of elements that are generated by other rules. The output of this rule is a polyline auxiliary element connecting the set of 3D points. The rule proceeds in three steps. First, an initial polyline is created by connecting the 3D points. For this purpose, the generator starts at a random point and connects it to a random point in close proximity until all points are connected. The resulting polyline may have strong kinks. To remove the kinks, a cubic interpolation and subdivision is applied resulting in a smoother polyline. The fiber structures that we would like to model are characterized through a persistence length property that expresses the bending stiffness of a fiber. Midpoint displacement is able to incorporate the target stiffness by increasing or decreasing the amount of displacement [38]. Therefore, in the third step, the midpoint displacement algorithm is used to enhance the curve with detailed windings.

## 6 Use case: SARS-CoV-2

The novel coronavirus SARS-CoV-2 is currently posing an international threat to human health. As with previous SARS and MERS outbreaks, it emerged through zoonotic transfer from animal populations. These types of emerging viruses pose a continuing threat, and the biomedical community is currently launching a widespread research effort to understand and fight these viruses. Understanding of the mesoscale structure will play an essential role in understanding the modes of interaction of these viruses with their cellular receptors and designing effective vaccines. We introduce the biology first and then describe our modeling strategy.

Our mesoscale models integrate a growing body of cryoelectron micrographic data on entire virions with atomic structures of the biomolecular components. SARS-CoV-2 contains four structural proteins, a single strand of genomic RNA, and a lipid-bilayer envelope [49]. Other non-structural and/or host proteins may also be incorporated into the virion—this is a topic of current study in the field and it is not addressed in these models. Three of the structural proteins are embedded in the membrane. The spike (S) protein extends from the surface and forms the characteristic spikes that give the viruses their crown-like shape, as seen by electron microscopy. The spikes recognize cellular receptors and mediate entry of the virus into cells. The membrane (M) protein has an intravirion domain that interacts with the nucleoprotein and is involved in packaging the viral genome as the virus buds from the infected cell’s surface. The envelope (E) protein is a small pentameric complex that forms an ion pore through the membrane, which is thought to be involved in the process of budding, with only a small number of copies being incorporated into the virus. The viral genome is a single strand of RNA about 30,000 nucleotides in length, which is one of the largest genomes of RNA viruses. It is packaged by the nucleoprotein (N), which coats and condenses the RNA strand. Detailed description of the individual protein models is presented in Supplementary Material.

Due to the rapid progress and numerous discoveries regarding the structure of SARS-CoV-2 since the beginning of the pandemic, our technique has faced frequent updates of the model. Thus, we created two main versions of the SARS-CoV-2 model, first in April 2020, labeled as *20-04* and the second in July 2020, labeled as *20-06*. The latter incorporates mainly new findings about S proteins [34]. The model revision demonstrates the fulfilment of the revisionability requirement.

We describe the modeling process in the remainder of this section. First, we create the model of virion and later, the RNA construction is presented.

### 6.1 Virion modeling

**Fig. 10.**
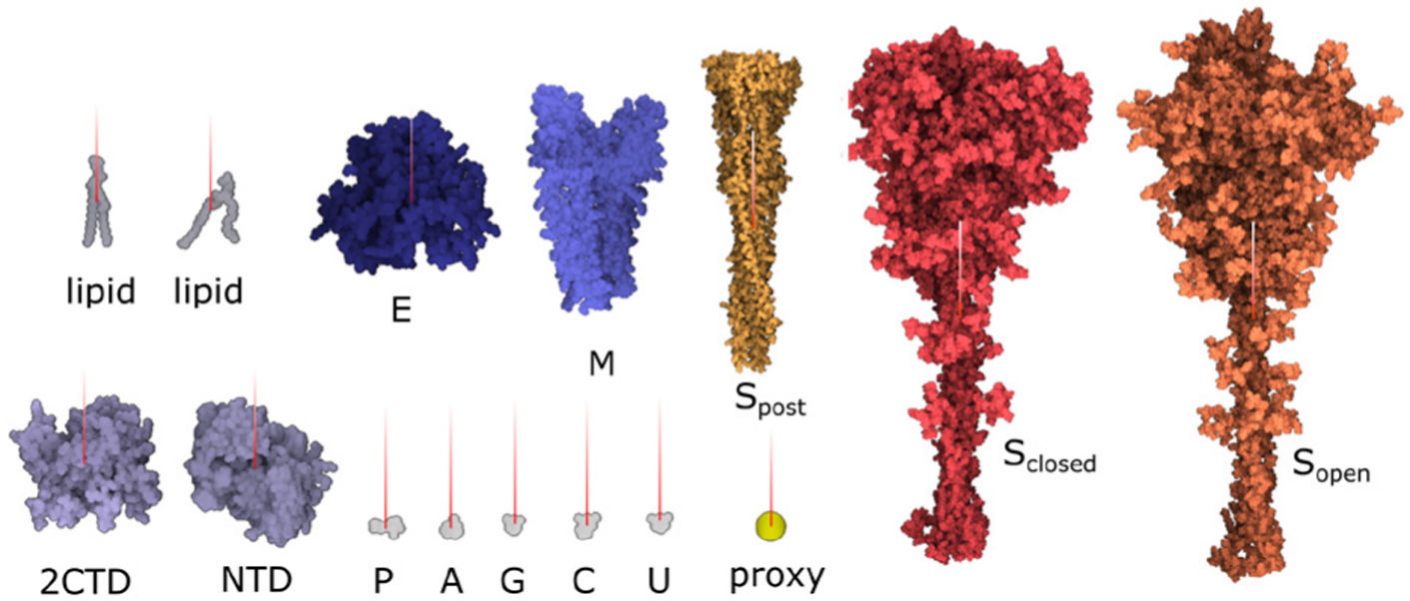
Setting of normal vectors to individual atomic structures.

Due to an arbitrary rotation of molecular models in the PDB files, we specify a normal vector for membrane-bound components to define their orientation and location within the membrane (see Figure 10). The whole modeling phase is performed by taking into account the estimated amount of individual elements published in [2] and [34].

We implemented a tool in which the user can segment 2D electron microscopy images (see Figure 3) and assign a real-world length. Firstly, the scale of the image has to be set. The tool then uses this scale throughout the entire segmentation process. Afterwards, the user manually segments the image and creates the outer contour by drawing a polyline enclosing the virion. After the outer polyline is finished, the inner contour is created by scaling down the outer contour. The scaling is driven by the user and can be updated whenever necessary. Once the inner contour is defined, the band between outer and inner contour is automatically subdivided into ten equally sized regions. As the last step, the user visually identifies the spikes in the image and marks them using proxy objects available in our utility. The tool automatically identifies the closest virion for the spike and assigns the spike into the corresponding contour region. After this assignment, the histogram is updated.

This outer contour and histogram are the input for the statistical determination (Section 4). We process the data and estimate a new contour, as described previously. From the newly generated contour, we create a 3D triangular mesh and assign each of its triangles the amount of containing S-protein instances based on the histogram sampling (see Figure 3).

**Fig. 11.**
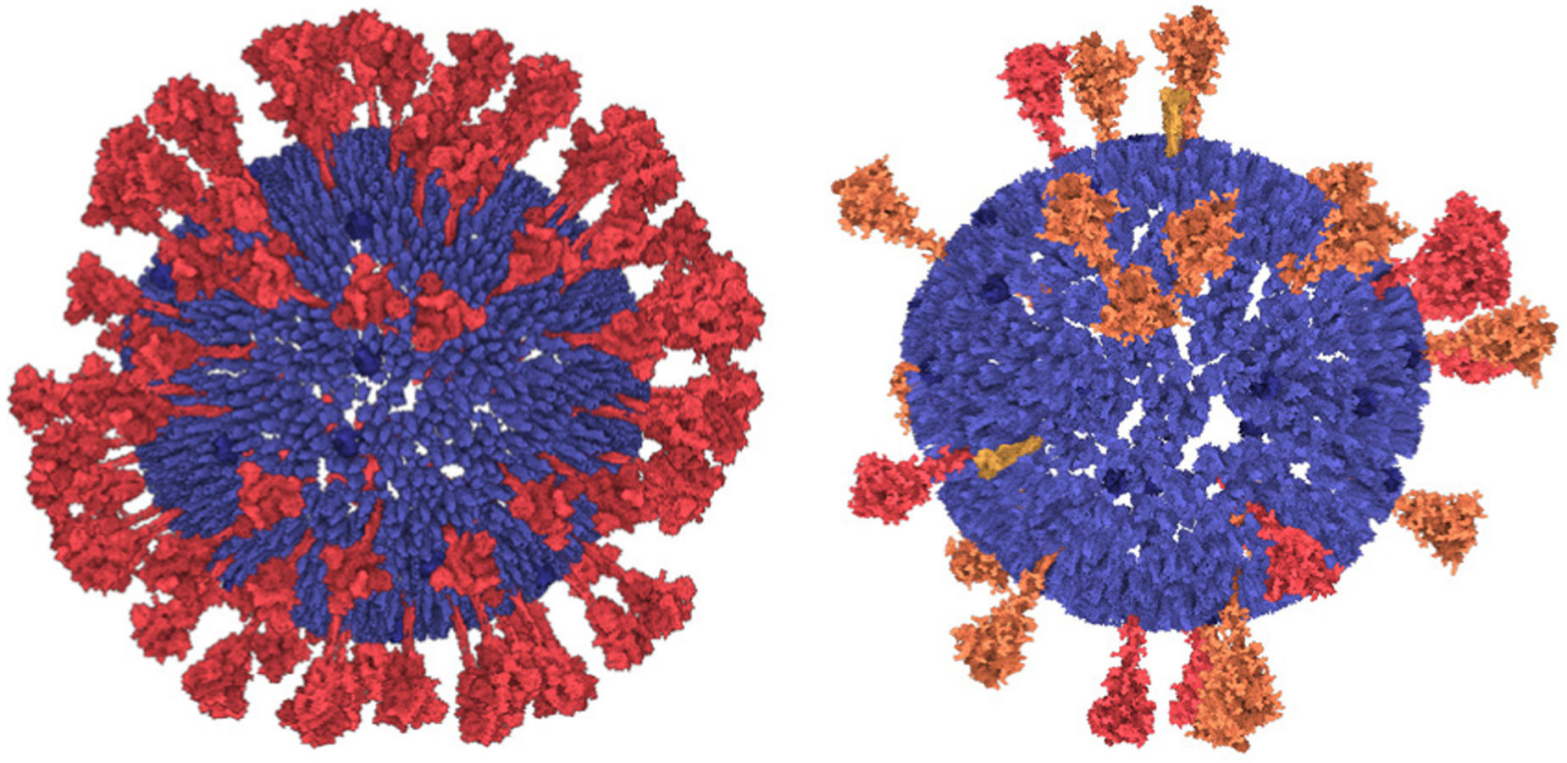
Spikes scattered on the surface of the 3D mesh according to the estimated distribution function. Membrane and envelope proteins are uniformly distributed on the membrane. Left: Model version 20-04. Right: Model version 20-06.

In the next step, **S proteins** are placed on the surface of the mesh. The user specifies the distance from the center point of a spike to the surface of the 3D mesh and the number of spikes to be placed. The parent-child rule (see Section 5.2) is used: the parent is the 3D mesh and children are the spikes. The illustration is depicted in Figure 11. For every type of spike protein (*S*_*open*_, *S_closed_*, *S*_*post*_), the rule differs in the number of instances and in the distance to the surface.

A similar rule is used for both **M and E proteins**. The only difference is that these protein instances are uniformly distributed, i.e., no amounts distributed over the 3D mesh based on observation on the contour are taken into account. These proteins are not discernible on the images and their distribution have not yet been characterized. For the time being, our model assumes a uniform distribution on the mesh(see Figure 11).

**Fig. 12.**
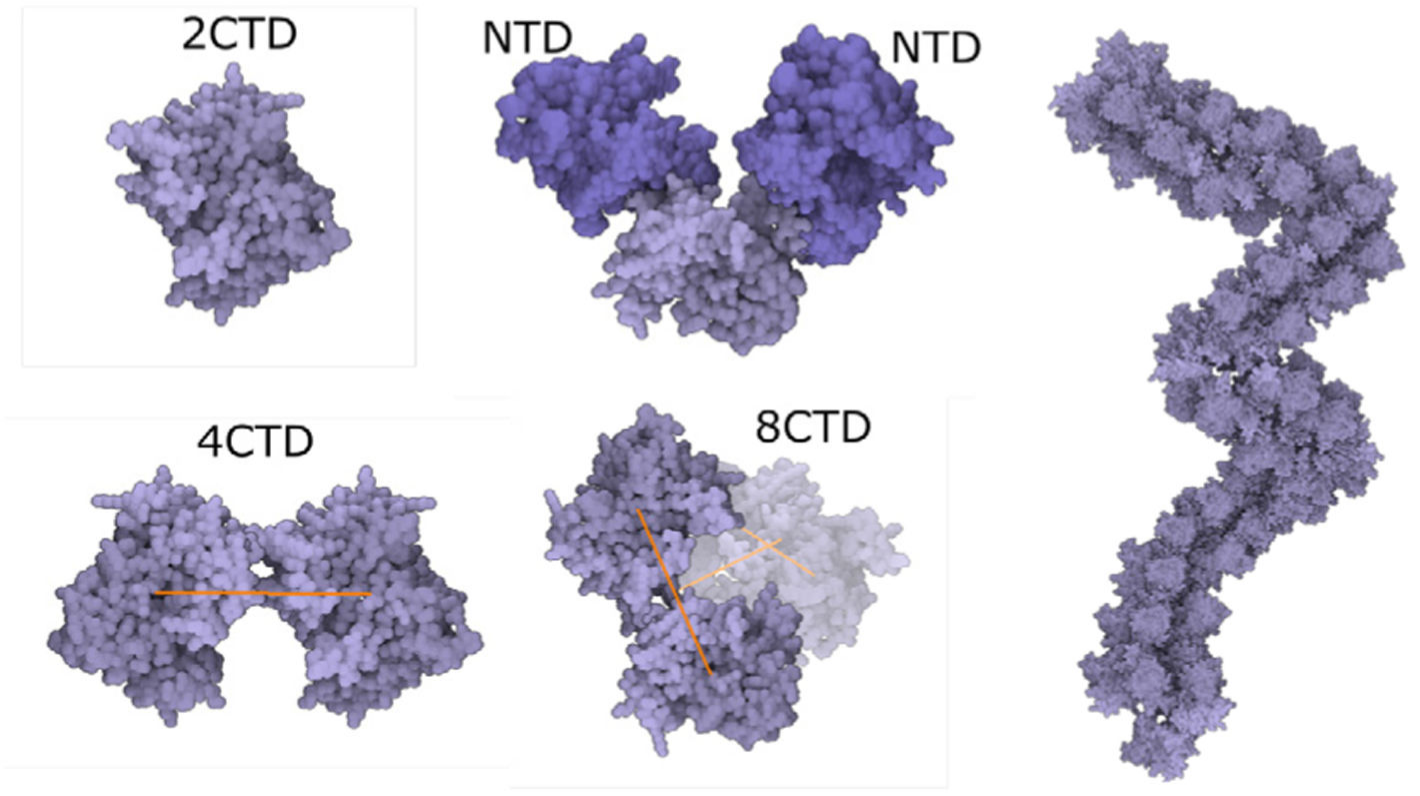
Rope-like N protein complex rule. Left: Creating an N protein CTD octamer structure and its relation with NTD. Right: Connecting N protein octamer structure to form the rope.

The **nucleoprotein complex** is built in several steps. To begin, a fiber-like assembly of N protein conformations is built. The N protein conformation is modeled using a dimer of the C-terminal domain (2CDT) and N-terminal domain (NTD) instances as follows. Firstly, a siblings rule is created to bind two NTD to 2CDT (see Figure 12 top-left). Although there are only two relations depicted in the image, we create the total amount of six relations between 2CTD and NTD. In the population phase, only two out of six created relations are randomly chosen. In the following step (see Figure 12 bottom-left), 2CTD is bound to rotated 2CTD using the siblings-parent rule to a linear skeleton. This forms a tetramer 4CTD. Repeatedly, 4CTD is bound using a linear skeleton to another instance of 4CTD, forming an octamer 8CTD. The final N protein assembly is constructed using the siblings-parent rule of 8CTD and a polyline skeleton. To create the polyline skeleton, we uniformly fill the interior of the 3D mesh with instances of a proxy object. This proxy object is a sphere that is customized to have a radius approximately the same size as the radius of the bounding sphere of 8CTD. By applying the connection rule Section 5.2 on such proxy spheres, we obtain the polyline skeleton. Finally, the siblings-parent rule is applied to 8CTD and the polyline forming the N protein assembly is created. RNA is then added to form the entire nucleoprotein complex, as described in the next section. Note that at this point we cannot use the approach of Klein et al. [38] for placement of the N-proteins (the rope) because this algorithm uses a predefined size of the block, which is embedded in the algorithm. At the beginning, our building blocks are of an unknown size; the user can create arbitrarily sized building blocks (a group of proteins) that are subsequently chained along the polyline. Therefore, we designed a more general approach.

**Fig. 13.**
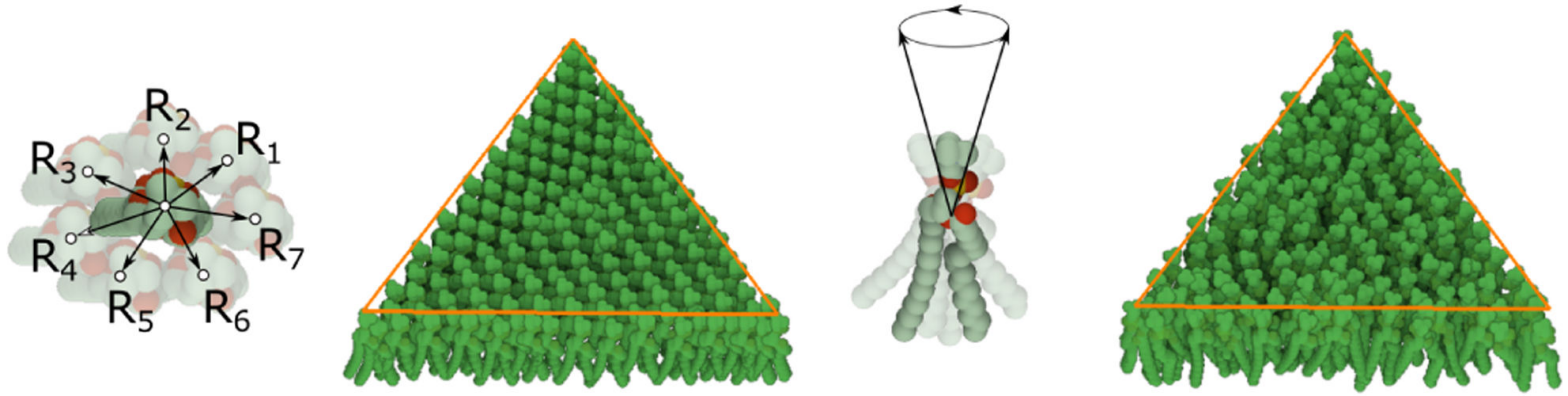
Population of lipids. Left: The rule with seven relations is created for a lipid. Application of the rule on triangular skeleton forming patterns. Right: Modifying the rotation of the lipid by setting yaw, pitch, roll. Resulting population on the triangular skeleton.

The **lipid bilayer membrane** is constructed using the siblings-parent rule. In reality, both layers can be modeled by the similar rule with the only exception that the lipids in one layer are rotated so that they are oriented to each other with their hydrophobic part. The construction of a single layer is as follows: Two copies of the same lipid model are added into the scene. The user creates a rule with several relations by translating (and rotating) one copy of the model to the desired position and storing these positions together with the distance to the 3D mesh parental skeleton. In our model, seven relations *R*_*i*_ are created (see Figure 13 left) for the transition from one lipid to another lipid. We use two models of different lipids - *Li*_1_ and *Li*_2_. Therefore, there are seven relations for each of the combinations: *Li*_1_ → *Li*_2_, *Li*_2_ → *Li*_1_, *Li*_1_ → *Li*_1_. The generating process starts in randomly chosen triangles of the 3D mesh parental skeleton. In the beginning, one lipid is added. In the next steps, up to seven new lipids can be generated. The rule is reapplied on these new instances. This process continues until 100 consecutive collision hits are accumulated, which indicates a fully dense lipid membrane. Note that only populating models with a limited amount of relations over the surface would lead to an occurrence of visible patterns. This is overcome by fine-tuning the rotation of newly placed elements by specifying how the standard deviations of yaw, pitch, roll vary (see Figure 13 right).

### 6.2 RNA modeling

**Fig. 14.**
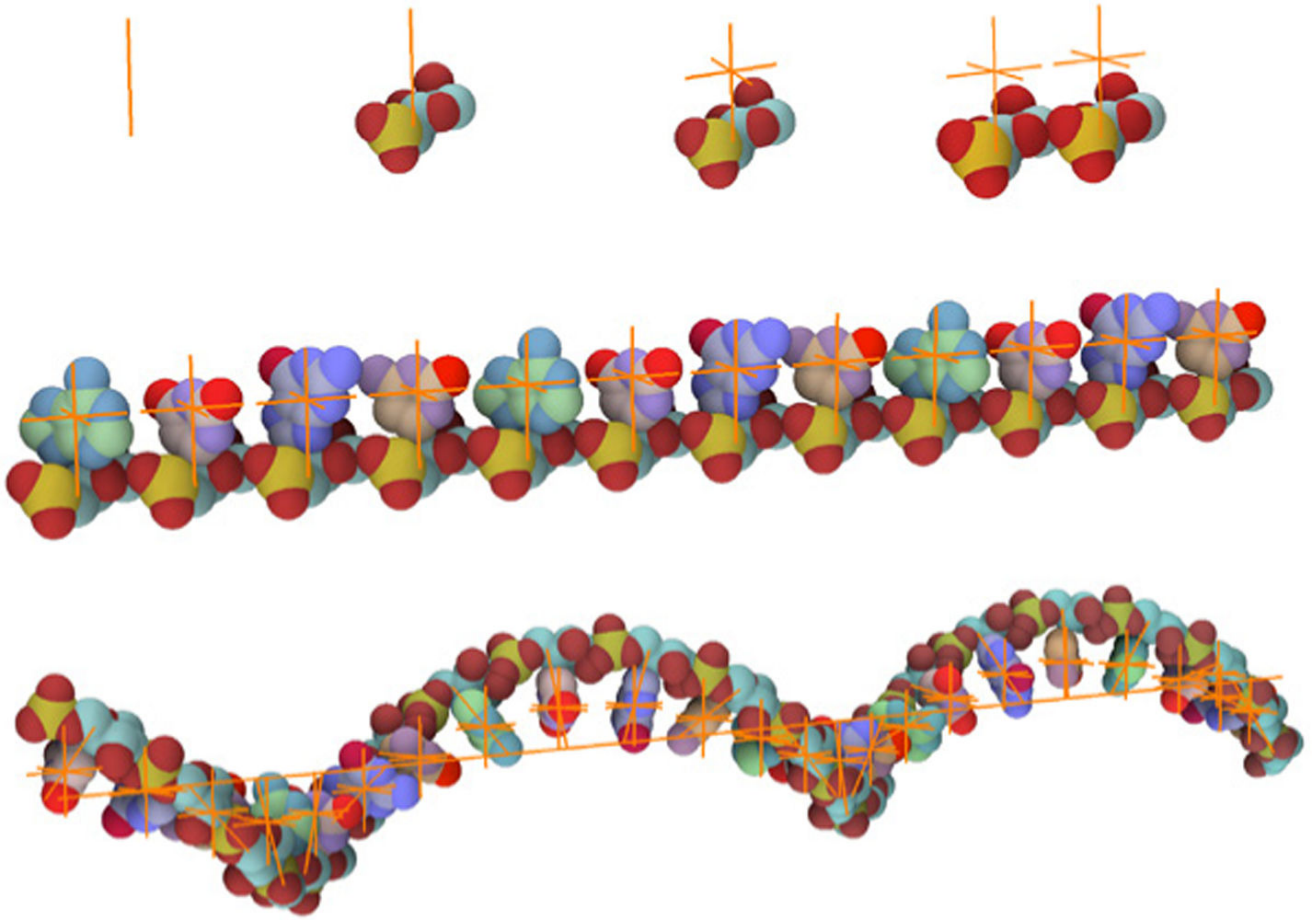
Illustration of RNA building. Top: Creation of a nucleotide and binding of two RNA nucleotides is illustrated (only the phosphate sugar backbone is shown; the bases will be populated at the point proxy above the backbone). Middle: The replication of the rule and replacing of proxies with A,C,G,U models. Bottom: The rule with a rotation incorporated applied to a line segment skeleton.

We have modeled RNA using five elementary models: four RNA bases adenine (A), cytosine (C), guanine (G), uracil (U), and one model consisting of phosphate and sugar (P) that forms the RNA backbone. A model of an individual nucleotide is created using the following approach. To a line skeleton using the parent-child rule, a P part and a point skeleton are bound (see Figure 14 top). The point skeleton in this case plays a role as a proxy object. In the population phase, a corresponding model of a base (A,C,G,U) from a genome string is placed to this position to finish the formation of the intact nucleotide. The genome string is specified by the user during the definition of the point skeleton rule.

In the next step, the siblings-parent rule of these individual nucleotides with a line skeleton is created. For simplicity, only the translation is presented in Figure 14 top-right. Once this relation is applied to a line skeleton, an RNA strand is created (see Figure 14 middle).

If a rotation between two nucleotides is specified during the process of modeling, the resulting RNA structure will twist along the line skeleton (see Figure 14 bottom). The consecutive bases with their phosphate sugar backbone forms the RNA string. Now the RNA model is created as a template and can be used anywhere that RNA is needed. However, the RNA model needs a skeletal structure along which it will form the RNA backbone fiber and then populate the bases according to the given RNA sequence. In our case, we replace the line skeleton with a polyline skeleton that represents a 3D curve connecting binding pockets of N proteins inside the core of the virion.

**Fig. 15.**
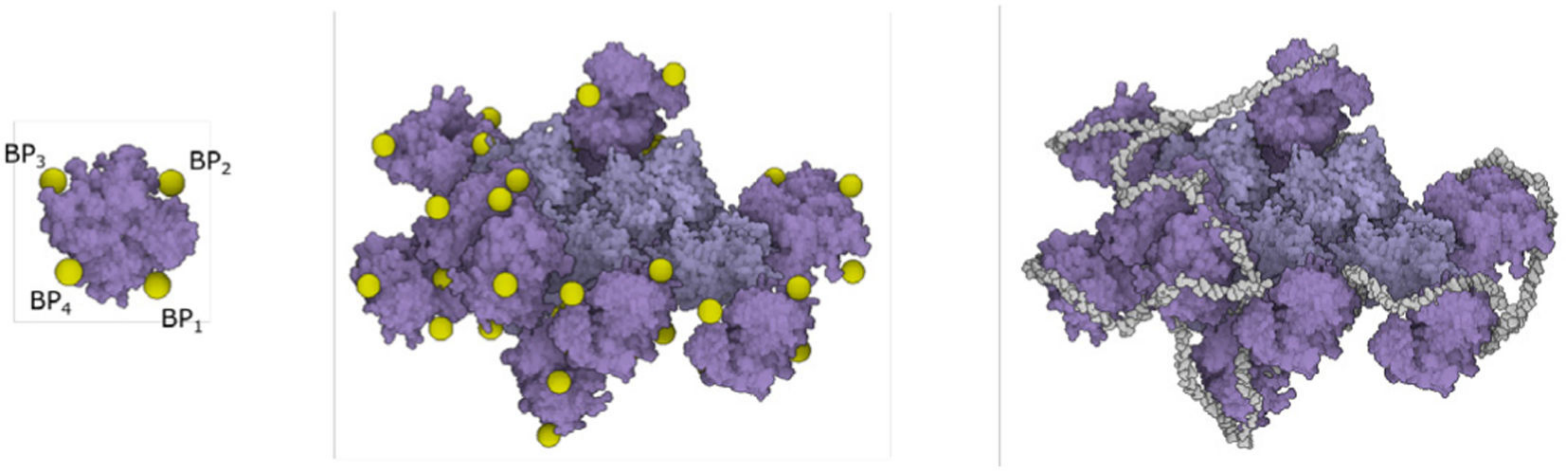
RNA proxy objects. Left: Specifying of the proxy objects representing RNA binding pockets on the surface of a few N proteins. Middle: The binding pockets computed on all N proteins in the model. Right: The resulting RNA after populating A,C,G,U,P along the 3D curve approximating the proxy objects.

In the final model, the RNA of the virion is bound to the predicted sites on the surface of N proteins. To specify the points on the N protein that define the RNA path, a rule with relations of a proxy object *BP*_*i*_ to N protein was created (see Figure 15 left). After populating the N proteins in the model, the algorithm computes positions of all proxies in the scene. The RNA backbone is obtained by using the connection rule and a 3D curve generator.

## 7 Discussion

The virion model is constantly undergoing many revisions as new information about its ultrastructure emerges and the literature is subsequently updated. Using standard modeling approaches, this dynamic situation with constant emerging information often necessitates a complete reassembly of the model. In our case, several rules needed to be redefined and an updated model was instantly generated. This real world experience, where a stream of new information is constantly being generated, has confirmed that our modeling framework is sufficiently versatile to accommodate new revisions with a given set of rules. Moreover, our modeling framework presents a rapid process for complex structural characteristics of a virion. The benefit of rule-based modeling is the nature of *templating,* which is advantageous for its ability to reuse the assembly patterns for other highly similar biological models. Therefore, for example, once the RNA rules are specified, they can be effortlessly applied in another model. If more structural knowledge comes to light or a more advanced model of the RNA is refined, our model can still be used in all mesoscale models that contain the RNA rule. The templating can be utilized, for example, in capsids, fibers, or membranes. This property inherently supports collaborative efforts, where modelers can revise the initial models of their peers, and a community can gradually build a large base of mesoscale biological assemblies. Additional models created with the same set of rules but based on different contours are presented in Appendix C.

Today, the availability of mesoscale models provides new opportunities to understand the structure and function of SARS-CoV-2. The number and distribution of spike proteins is still a matter of some conjecture; however, this information is relevant to fully understand the interactions of the virus with its cellular receptors and its interaction and neutralization by antibodies. The details of nucleoprotein condensation and packaging through interaction with the viral membrane proteins are also of interest because they provide possible targets for therapeutic intervention.

Currently, our entire approach is implemented as a single-threaded application on the top of the Marion molecular visualization framework [45] and, as a proof-of-concept implementation, it is not significantly optimized for performance. Some processing stages are not calculated at an instance. However, we believe that the overall user experience is sufficiently performant for the rapid prototyping of biological mesoscale models. The resulting model consists of 29 S (in different states), 1000 M dimers, 25 E, ~1000 N (in N-CTD and N-NTD), 29903 bp ss-RNA bases (GenBank: MN908947.3 [79]), and 29903 P elements forming the RNA backbone, and ~180000 lipids. The entire model is created by 23 rules (with 59 relations) defined by the user. Several of these are different possible configurations of the same elements (as in the case of lipids). The population of S, E, M, N, and all parts of RNA are processed within 2 seconds each. The population of lipids is the most computationally demanding part of the algorithm, taking approximately two minutes for each inner and outer membrane, primarily because there are many lipid samples that are regressed. However, the required time is heavily dependent on the rules defined. We have created a very dense distribution of lipids with five relations for the rule.

Our technique can be generalized on three levels: system level, whole-cell level, and molecular level. First, at the system level, our technology can be applied not only for biological objects but also objects in materials, chemistry, and physics, i.e., wherever instances of the same or similar structural elements form hierarchies by assembling into a complex structure. Second, at the whole-cell level, our technology can currently extract the overall shape of biological objects that have a star-domain property, i.e., there is at least one point inside, from which all internal points are directly *visible,* without crossing the contour. This includes many viral envelopes, capsids, and compact cellular compartments. Finally, at the molecular level, our system can generalize the model for many types of viruses and simple bacteria; however, with their molecules without any motion.

These generalizations simultaneously define the scope and limitations, i.e., the system in its current form is missing a complex compartmental specification, such as the inner mitochondrial membrane, endoplasmic reticulum, or Golgi apparatus, for example. While it is possible to model with very high complexity, modeling an asymmetric complex, such as the HIV capsid, will impact on the speed of the modeling process because a non-trivial amount of rules and effort are required as an input from the user. Another limitation is the case of *sticky* fibers, such as single-stranded genome macromolecules, which often form complex secondary structures that are enabled through sequence complementarity. It is still unclear whether such a characterization is possible to be expressed using our system, or if we would need to expand the rule set. Finally, the entire model does not integrate any notion of emergent behavior.

## 8 Conclusion and Future Work

In this paper, we have presented a new system for the rapid modeling of mesoscale biological models. Challenged with frequent revisions of the SARS-CoV-2 model, we demonstrated that our framework is suitably versatile and is able to incorporate any new structural insights. The benefits of this work for the scientific community are two fold: we present a new technology and a new structural model of SARS-CoV-2^1^ that might lead to the development of effective vaccination or treatment strategies. There are several research questions that are difficult to answer without explicitly imaged evidence. One such question is: How many copies of RNA can a single virion pack? Is it just one or is there possibly enough space to accommodate another copy? The utility potential for hypothesis generation of biological questions that are of an integrative structural nature is tremendous.

This framework allows for varying levels of model precision. A model can be specified exactly so that one amino acid interacts with another, or it can be placed more roughly. The system of rules preserves the accuracy, which is given as input. Combined with the specification of flexibility and collision handling, we can achieve even simple geometric docking.

In the future, we plan to extend this method to generate the overall shape of the virion from more cross-sections or volume data. We also plan to incorporate surface representation from virion contours that would estimate spherical-harmonics coordinates to replace the contour inflation approach. Modelling asymetric HIV capsid can be made quicker if additional rule concepts are introduced. For example, a new type of *tessellation* rule, based on Euler’s characteristic for convex polyhedra, would constrain the specification on how a 3D object is to be formed by various building elements. Two rule-based modeling systems Biogen [26] and Kappa [6] show promise for expressing the interaction patterns and we aim to complement kinetic or agent-based systems with our modeling technique.

It is interesting to study the interplay of rule-modeled structures and reconstruct details from microscopic images that are difficult to discern by fitting a particular rule-expressed pattern into the image. Parallelizing our implementation would allow for interactive performance, where a large number of conformations can be tested within a short time. Iteratively fitting the detail to an unclear microscopy image may be a way to solve an inverse problem in a brute-force manner.

Modeling with mouse interactions can be complemented with advanced speech recognition, to allow for voice-controlled modeling. Simultaneously, the ontology community has created a rich categorization of shapes that have, however, no associated geometry. By establishing such an association through the usage of terminology in shape ontologies, a verbal specification of models can lead to the desired model.

## Supplementary Material

Suppl Video

Suppl File

## Figures and Tables

**Fig. 1.**
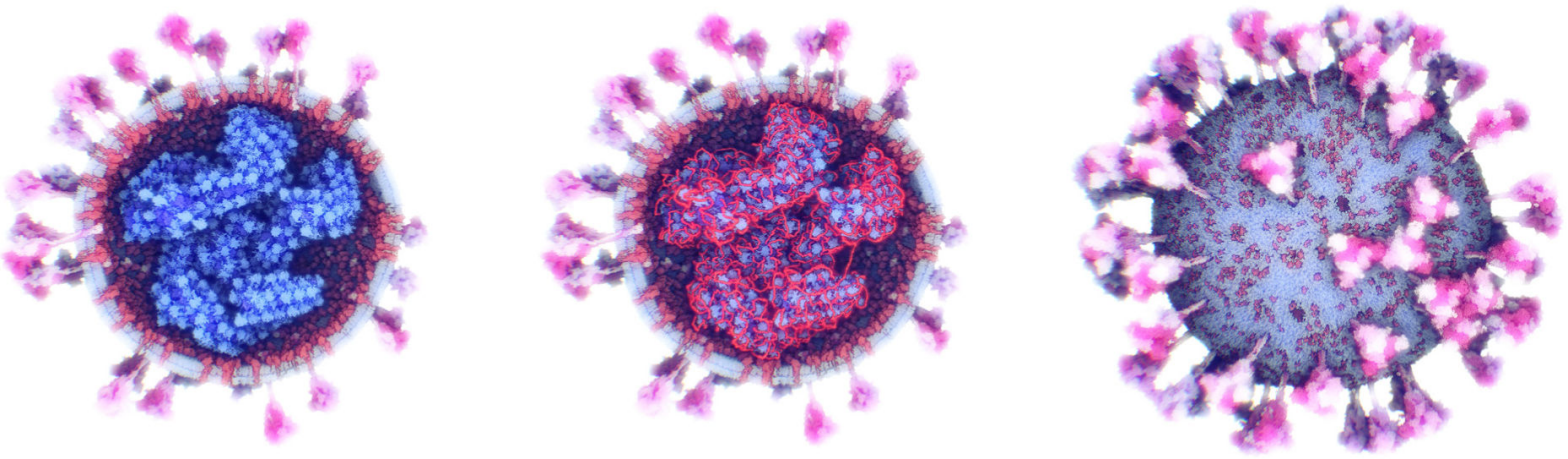
The ultrastructure of a SARS-CoV-2 virion created using our modeling technique. The membrane shape and distribution of the spike proteins are determined from microscopy image data. The internal assembly is a result of an interactive 3D rule specification approach. Left: internal *nucleoprotein* complex. Middle: RNA condensed within the nucleoprotein. Right: Outer spike distribution.

## Footnotes

1 available at nanovis.kaust.edu.sa/sars-cov-2-virus-model/
