# Supplementary material for: Modeling in the Time of COVID-19: Statistical and Rule-based Mesoscale Models
Source: IEEE Trans Vis Comput Graph. Author manuscript; Available in PMC 2021 Dec 4. (PMC8642830; doi:10.1109/TVCG.2020.3030415)
Supplement: Supplementary file 1 [file NIHMS1755333-supplement-Suppl_File.pdf]

SUPPLEMENTARY MATERIAL TO THE SUBMISSION #1175

A ALGORITHMS AND DATA STRUCTURES

Algorithm 1: Generate new contour from extracted contours.

```
GenerateContour (C, N_sample)
    Input  : Contours set C; # angle directions N_sample
    Output : Generated contour C_g
    //Augmented data
    C_stat = [];
    foreach c ∈ C do
        cp = c.Rotate(π);
        cf = c.Flip(x-axis);
        cpf = cp.Flip(x-axis);
        C_stat.add(c, cp, cf, cpf);
    end
    //Get max distance from all points on contours to origin
    R_stat = [];
    angle_sample = [];
    foreach c ∈ C_stat do
        foreach point p ∈ c do
            R_stat.add(Distance(p, origin));
        end
    end
    r_max = Max(R_stat);
    //Cast a ray from origin to all directions
    i = 0;
    while i++ < N_sample do
        angle_sample.add(2iπ/N_sample);
    end
    Ray_sample = array(N_sample);
    i = 0;
    while i++ < N_sample do
        Ray_sample[i].x = r_max cos(angle_sample[i]);
        Ray_sample[i].y = r_max sin(angle_sample[i]);
    end
    // Fit normal distribution of r_∩ for each angle
    foreach r ∈ Ray_sample do
        intersections = [];
        foreach c ∈ C_stat do
            intersections.add(Intersect(r, c));
        end
        r_∩ = Distance(Intersect(r, c), origin);
        σ_s = StandardDeviation(r_∩);
        μ_s = Mean(r_∩);
        min_s = Min(r_∩);
        max_s = Max(r_∩);
    end
    //Rejection sampling for each angle
    C_g = [] foreach θ_s ∈ angle_sample do
        C_g.add(RejectionSampling(θ_s, min_s, max_s, μ_s, σ_s));
    end
    //Interpolate C_g
    C_g = CatmullRom(C_g);
```

Algorithm 2: Rejection sampling for create new contour.

```
RejectionSampling (θ_s, min_s, max_s, μ_s, σ_s)
    Input  : Angle θ_s;
    distribution of r_∩ of θ_s: min_s, max_s, μ_s, σ_s
    Output : One sample point of new contour p_g
    accept = false;
    while !accept do
        Choose r ~ U(min_s, max_s);
        Choose prob ~ U(0, MaxProbabilityDensity(r_∩));
        pdf(r) = ProbabilityDensity(r);
        if pdf(r) ≤ prob then
            accept = true;
            p_g.x = r cos(θ_s);
            p_g.y = r sin(θ_s);
        end
    end
```

Data structure 2: Description of a rule r data structure.

```
struct {
    string Name_in, Name_out; // identifier of elements
      E(Name_in), E(Name_out)
    int count; // temporary counter of generated elements
    int count_max; // number of elements to be placed
    enum distanceDistributionType // type of distribution
      function used [Uniform, Gaussian]
    int collisions; // temporary counter of detected collisions
    int collisions_max; // maximal number of errors while
      collisions computation
    string groupName; // what group the rule belongs to
    float probability; // user-defined probability
    int T_num; // number of transformations processed
    float yaw|pitch|roll; // user-defined maximal allowed
      deviation in rotation
    float T_i.distance; // distance of E(Name_in) to E(Name_out)
    float T_i.distanceStdDev; // standard deviation of a
      generated distance (default=0)
    float T_i.probability; // probability of selecting the
      transformation
    quat T_i.rotation; // rotation of E(Name_out)
    vect T_i.translation; // translation from E(Name_in) to
      E(Name_out)
    quat T_i.angularDifference; // rotation from vector
      (S, E(Name_in)) to vector (S, E(Name_out)) if bound to a
      skeleton S
    int T_i.v_j; // closest vertex of the bound skeleton
} Rule;
```

B Evolution of the model in time

This section contains the biological information about SARS-CoV-2 that we used during the model creation phase. Both of the subsections describe the current state with the respect to the time the text was created. Thus, these are two versions of the same text. Firstly, the text from April 2020 is presented (model version 20-04). Since that time, a huge amount of research on SARS-CoV-2 has been done worldwide. Therefore, we present a revised version that we developed in June 2020 (model version 20-06). We want to keep both these versions here not only because of the model itself, but rather to demonstrate that new information regarding the model can be revealed within months and at a very fast pace. Thus, the ability to process this information in a rapid manner is crucial.

B.1 SARS-CoV-2 v.20-04

At the initiation of this project, structural information about SARS-CoV-2 proteins was limited; thus, we employed homology modeling to generate atomic structures of the viral proteins. The automated pipeline described here is created so that the most current structural models may be substituted as additional information emerges. This project also made extensive use of rapid modeling capabilities to incorporate new sources of data as they became available.

The *spike protein* is highly dynamic, undergoing several structural transitions in the process of viral entry. In the free virion, most of the spike proteins are in the "prefusion" state. This will transition to a fusion-competent state when the virus encounters a cell. Several structures of the S protein in its prefusion state have been determined with cryoEM (PDBids: 6VSB, 6VXX, 6VYB). These structures cover most of the protein, leaving out about 130 residues at the C-terminus, corresponding to HR2, transmembrane, and cytoplasmic domains. The N protein has been partially determined with X-ray crystallography (PDBids: 6VYO, 6YI3, 6WJI, 6M3M). As for SARS-CoV-2 M and E protein; however, none of them have been experimentally resolved yet. In this work, we took advantage of SARS-CoV-2 sequence homology with other betacoronaviruses to perform homology modeling and to obtain a structural model for each protein component present in the SARS-CoV-2 virions.

The S protein model was obtained by a composite homology modeling approach. Residues 27-1146 were modeled with SWISS-MODEL [74] using 6VXX as a template to reconstruct all the missing loops that were not solved in the experimental structure. Residues 1147-1212, 1213-1242, 1243-1273 corresponding to HR2, transmembrane, and cytoplasmic domain, respectively, were submitted separately to the homology modeling server for oligomeric structures GalaxyHomomer [1]. Template-based modeling generated the HR2 structural model, utilizing the structure of MERS-CoV fusion core (PDBid 4MOD, 39% sequence identity) as the template, while the transmembrane and cytoplasmic models originated from ab-initio modeling methods. The structures of the four components were aligned in PyMol [63] and merged to obtain a unique model in Coot [18].

Pentameric *E protein* was modelled with the protein structure prediction server Robetta [36] in 'comparative modeling' mode. The NMR structure of E protein from SARS-CoV (PDBid 5X29) was used as the template, as the two proteins share 94% of their amino acid sequence. The structural model for M protein (residues 11-203) was predicted by DeepMind using AlphaFold prediction methods and freely distributed in response to the COVID-19 crisis [33]. Residues 3-10 were built onto the original model with Coot [18] and a N-glycosylation was added to residue N5 through GLYCAM-Web [66].

The structure of the viral *nucleoprotein complex* is a topic of current speculation and study, so we created a model that is consistent with estimates of the number of copies of N, the length of the genome, and the locally ordered arrangement of proteins as seen by cryoelectron microscopy [50]. N protein folds to form two stable globular domains, connected by a flexible linker. The C-terminal domain (CTD) forms dimers, as seen in PDBid 6WJI of the SARS-CoV-2 CTD. In the SARS CTD, these dimers may further associate into octamers, as seen in PDBid 2CJR. The structure of the N-terminal domain (NTD) has been determined in PDBid 6YI3, and is often termed the RNA-binding domain, although RNA binds to multiple sites in the N protein [35]. In cryoelectron micrographs, the nucleoprotein complex is seen to have short-range order as a close-packed collection of spherical features closely opposed to M protein on the inner face of the membrane [50], and gently isolated nucleoprotein complexes have the look of rope-like collection of beads roughly 15 nm wide [44]. These data have been integrated into a conceptual model where CTD octamers stack loosely into long ropes and NTD are loosely associated around the central rope, interacting with RNA [24]. We have implemented this conceptual model, first building CTD octamers, then placing NTD, and finally building a continuous RNA strand that winds through the NTD. Explicit models for the intrinsically disordered linkers and flexible portions of the N chain at the N- and C-termini will be the subject of a future study. The visualization of used structures and the model 20-04 can be seen in Figure 16.

B.2 SARS-CoV-2 v.20-06

At the initiation of this project, structural information about SARS-CoV-2 proteins was limited, so we employed homology models to represent atomic structures of the viral proteins. The automated mesoscale modeling pipeline described here is created so that the most current structural models maybe substituted as additional information emerges. The project also made extensive use of the rapid modeling capabilities to incorporate new sources of data as they became available.

A wealth of experimental structures for the *S protein* in its prefusion state have been determined by cryo-EM [8, 9, 13, 27, 30, 56, 72, 77, 78] et al. Two main conformations have been described, open or 'up' and closed or 'down', which refer to the receptor binding domain (RBD) orientation. These structures cover most of the S protein, leaving out about 130 residues at the C-terminus, corresponding to HR2, transmembrane, and cytoplasmic domains. Further, the most C-terminal part of the protein, including large parts of the stalk and the entire transmembrane domain, remained resolved at low resolution in any of the previous studies. The N protein has been partially determined with X-ray crystallography (PDBids: 6WKP, 6WZO, 6YUN, 6VYO, 6WJI, 6ZCO, 7C22, 6YI3, 6M3M). As for SARS-CoV-2 M and E protein, none of them have been experimentally resolved yet.

In this work, full length models for the prefusion S protein were generated by Casalino et al. [10]. Such models were reconstructed starting from solved cryo-EM structures (PDBid 6VXX for the closed state, PDBid 6VSB for the open state), supplemented with homology modeling for the unsolved HR2, transmembrane, and cytoplasmatic domains. Additionally, in these models the complete glycosylation pattern described for SARS-CoV-2 S protein [65, 73, 82] et al. was fully reconstructed.

Several studies have shown that a small portion of the S proteins decorating the SARS-CoV-2 virion appear in their post-fusion state [8, 70, 80] et al., which is the conformation S protein acquires upon host interaction and proteolytic cleavage. Experimental structures for the post-fusion S protein have recently been solved but, at the time of writing, have not been released yet [8]. The cryo-EM structure of SARS-CoV S protein have been used instead (PDBid 6M3W), which shares 90% of its sequence homology with SARS-CoV-2 for this specific region on the protein.

Pentameric *E protein* was modelled with the protein structure prediction server Robetta [36] in 'comparative modeling' mode. The NMR structure of E protein from SARS-CoV (PDBid 5X29 [67]) was used as template, as the two proteins share 94% of their amino acid sequence.

As of July 2020, no experimental structure for *M protein* is available. Therefore, the structural model for M protein (residues 1-222) was predicted via homology modeling by Micheal Feig's group [28] and freely distributed in response to the COVID-19 crisis. The M protein model was assembled as a homodimer [51] and subjected to refinement in a ER-like membrane environment. Finally, N-glycosylation was added to residue N5 of each monomer through GLYCAM-Web [66].

The structure of the viral *nucleoprotein complex* is a topic of current speculation and study, so we created a model that is consistent with estimates of the number of copies of N, the length of the genome, and the locally ordered arrangement of proteins as seen by cryo-EM

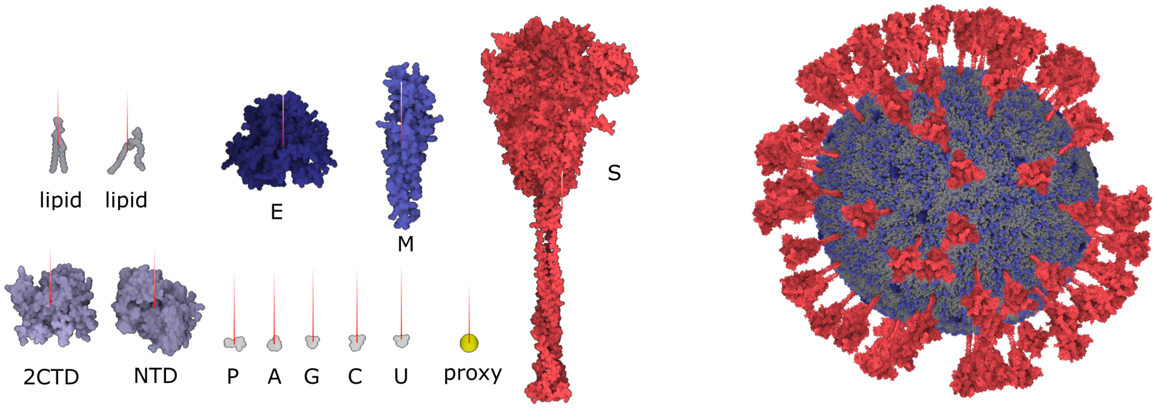

Fig. 16. Left: List of used elements. Right: The resulting model SARS-CoV-2 v. 20-04

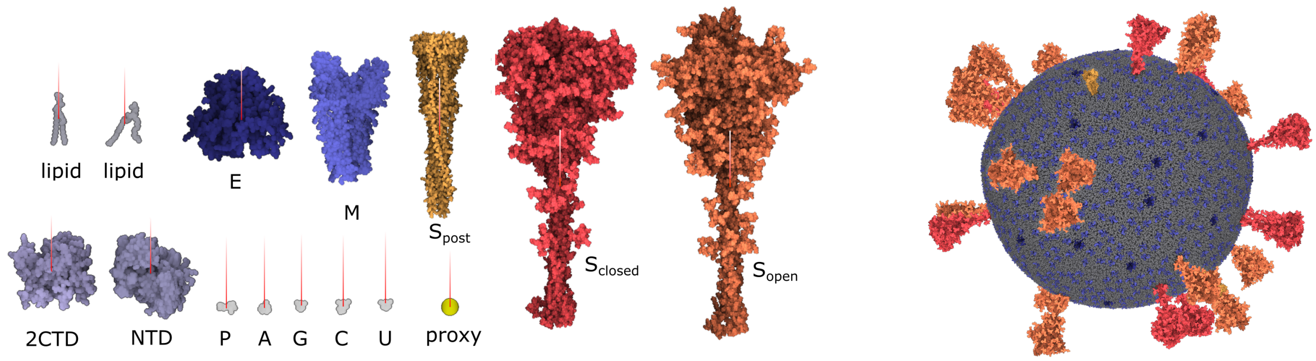

Fig. 17. Left: List of used elements. Right: The resulting model SARS-CoV-2 v. 20-06

microscopy [50]. N protein folds to form two stable globular domains, connected by a flexible linker. The C-terminal domain (CTD) forms dimers, as seen in PDBid 6WJI of the SARS-CoV-2 CTD. In the SARS CTD, these dimers may further associate into octamers, as seen in PDBid 2CJR. The structure of the N-terminal domain (NTD) has been determined in PDBid 6YI3, and is often termed the RNA-binding domain, although RNA binds to multiple sites in the N protein [35]. In cryo-EM micrographs, the nucleoprotein complex is seen to have short-range order as a close-packed collection of spherical features closely opposed to M protein on the inner face of the membrane [50], and gently isolated nucleoprotein complexes have the look of rope-like collection of beads roughly 15 nm wide [44]. These data have been integrated into a conceptual model where CTD octamers stack loosely into long ropes and NTD are loosely associated around the central rope, interacting with RNA [24]. We have implemented this conceptual model, first building CTD octamers, then placing NTD, and finally building a continuous RNA strand that winds through the NTD. Explicit models for the intrinsically disordered linkers and flexible portions of the N chain at the N- and C-termini will be a subject for future study. The visualization of used structures together with the final model can be seen in Figure 16.

C SARS-CoV-2 MULTIPLE MODELS

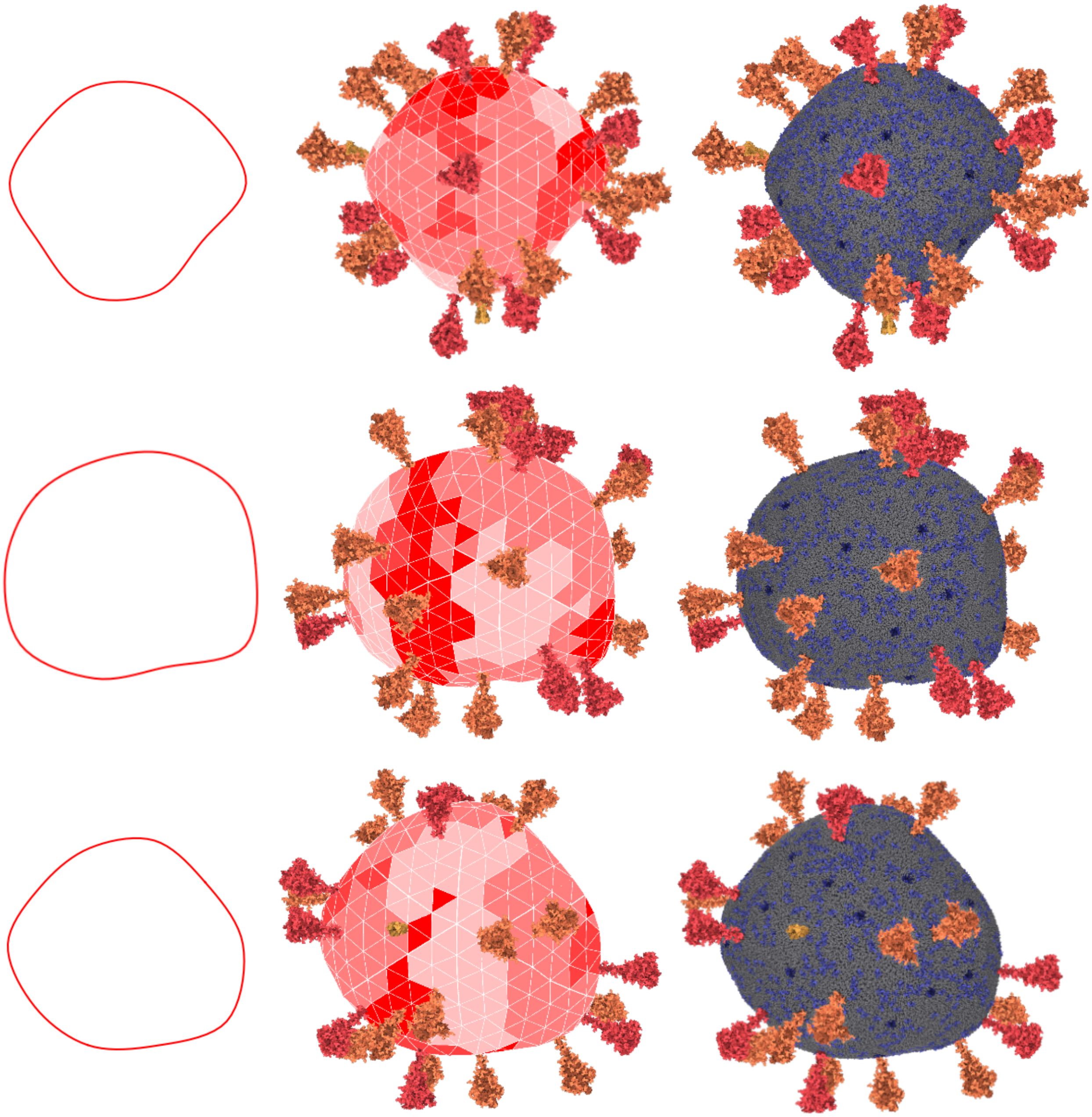

Fig. 18. With our approach, multiple models can be generated within a reasonable time. This image presents three other models generated using the very same set of rules. Left: Generated contours. Middle: The surface evaluated by the estimated distribution. Right: Final model.
